# Toric Spines at a Site of Learning

**DOI:** 10.1523/ENEURO.0197-19.2019

**Published:** 2020-01-02

**Authors:** Daniel Sanculi, Katherine E. Pannoni, Eric A. Bushong, Michael Crump, Michelle Sung, Vyoma Popat, Camilia Zaher, Emma Hicks, Ashley Song, Nikan Mofakham, Peining Li, Evan G. Antzoulatos, Diasynou Fioravante, Mark H. Ellisman, William M. DeBello

**Affiliations:** 1 Center for Neuroscience, University of California, Davis, CA 95618; 2 National Center for Molecular Imaging Research, University of California, La Jolla, CA 92093

**Keywords:** auditory processing, connectomics, dendritic spine, integration, neural circuit, synapse

## Abstract

We discovered a new type of dendritic spine. It is found on space-specific neurons in the barn owl inferior colliculus, a site of experience-dependent plasticity. Connectomic analysis revealed dendritic protrusions of unusual morphology including topological holes, hence termed “toric” spines (*n* = 76). More significantly, presynaptic terminals converging onto individual toric spines displayed numerous active zones (up to 49) derived from multiple axons (up to 11) with incoming trajectories distributed widely throughout 3D space. This arrangement is suited to integrate input sources. Dense reconstruction of two toric spines revealed that they were unconnected with the majority (∼84%) of intertwined axons, implying a high capacity for information storage. We developed an *ex vivo* slice preparation and provide the first published data on space-specific neuron intrinsic properties, including cellular subtypes with and without toric-like spines. We propose that toric spines are a cellular locus of sensory integration and behavioral learning.

## Significance Statement

The majority of excitatory synapses in the brain are formed onto dendritic spines, which typically act to isolate the action of individual synapses. We discovered a new type of spine which in contrast receives convergent input from many different axons. These “toric” spines, named for their topological holes, are found on space-specific neurons in the barn owl auditory system. We used a combination of *in vivo* electrophysiology, super-resolution optical microscopy and serial block-face scanning electron microscopy (SBEM) to characterize the ultrastructure and wiring of toric spines, and *ex vivo* patch clamp recording to elucidate the cells’ electrical properties. These data lead us to propose that toric spines serve as microanatomical hubs for neuronal computation, plasticity and learning.

## Introduction

Neuronal computations are shaped by postsynaptic morphology (Rall, 1974). Across circuits and species, dendritic spines receive the majority of excitatory synaptic input (Hering and Sheng, 2001). Most spines have narrow necks that serve to compartmentalize, electrically (Yuste, 2013; Kwon et al., 2017) and biochemically (Müller and Connor, 1991; Sabatini et al., 2001), a small number of synaptic inputs - usually one. In this arrangement the dendritic branch performs the first stage of integration of inputs coming from multiple sources (Poirazi et al., 2003). Here, we identify an alternative motif involving first stage integration via a new type of dendritic spine.

Space-specific neurons in the barn owl inferior colliculus (Knudsen and Konishi, 1978) compute sound source direction via integration of binaural cues, interaural time difference (ITD) and interaural level difference (ILD), and the elimination of phase ambiguity by convergence across frequency channels (Wagner et al., 1987; Olsen et al., 1989; Brainard et al., 1992; Konishi, 2003; Singheiser et al., 2012), computations known to involve multiplication and non-linear thresholding, respectively (Peña and Konishi, 2000, 2001, 2002, 2004). In addition, the responses of space-specific neurons can be modulated by visual input (Bergan and Knudsen, 2009) and attention (Winkowski and Knudsen, 2006; Mysore and Knudsen, 2014). Thus, space-specific neurons act as pattern detectors via the integration of complex sensory inputs, and make response adjustments based on behavioral context.

Space-specific neurons provide an opportunity to understand how neuronal computations are shaped by microanatomical patterns of synaptic convergence, but their ultrastructure is unknown. To fill this gap we used serial block-face scanning electron microscopy (SBEM; Denk and Horstmann, 2004) and stimulated emission depletion (STED) microscopy (Hell, 2003). Space-specific neurons in young adult owls were labeled *in vivo* by focal injection of retrograde tracer at functionally defined map locations (Rodriguez-Contreras et al., 2005). STED imaging revealed two cellular subtypes, one studded with typical spines and the other with a previously unreported type of spine with unusually complex and variable morphology, termed “toric” spines. SBEM imaging was used to reconstruct all 76 toric spines found on the soma and proximal dendrites of one space-specific neuron, along with their associated presynaptic terminals. In length, cytoplasmic volume and innervation density, toric spines resemble thorny excrescences found on pyramidal cells in mammalian hippocampus and amygdala (Amaral and Dent, 1981). Thorny excrescences receive numerous active zones (Chicurel and Harris, 1992) coming from one or at most two input sources (Wilke et al., 2013; e.g., individual mossy fibers in the hippocampus), forming the so-called detonator synapse (Urban et al., 2001). We found that toric spines receive a comparable number of active zones, but in contrast, these come from up to 11 different inputs. The latter arrangement is suited for integration, not detonation nor compartmentalization.

To date, knowledge of space-specific neuron function derives from extracellular recordings (numerous studies) and three reports using intracellular sharp electrodes (Peña and Konishi, 2001, 2002, 2004). Neither technique provides information on intrinsic electrical properties or can resolve individual synaptic events. We performed *ex vivo* patch-clamp recordings from putative space-specific neurons in brain slices from juvenile owls. Many of these cells exhibited large, atypical spines including toric-like structures, the apparent developmental precursors to mature toric spines. In total, these findings and newly established methods provide a path to investigate the causal connections between microanatomical structure and the neuronal computations that underlie high-level pattern detection.

## Materials and Methods

### Animals

A total of 12 barn owls (*Tyto alba*) of either sex were used in this study. Adult animals (>250 d old) were group-housed in large flight aviaries while juveniles (35–40 d) remained in isolated nest boxes with parents and siblings. All husbandry and experimental methods were approved by the University of California Davis Institutional Animal Care and Use Committee.

### Microelectrode recording and retrograde labeling *in vivo*

Four adult owls were used for *in vivo* retrograde labeling. Owls were anesthetized using 2% isofluorane, a mixture of nitrous oxide and oxygen (1:1) and wrapped in soft cloth. Craniotomies were opened above the tectal lobes and the owl was secured in a stereotax located inside a soundproof recording room. Insulated tungsten recording electrodes ∼6-MΩ impedance (FHC) were lowered through the forebrain to the optic tectum (OT) based initially on stereotaxic coordinates (17 mm rostral to the neck muscle insertion, 6 mm lateral to the midline) and then refined on the basis of response criteria. Visual receptive fields were measured by computer-controlled projection of static or moving dots onto a calibrated screen and the responses were used to navigate to the deep layers of OT (dOT; in 5/6 injections) based on characteristic latencies, preference for negative contrast stimuli, and absence of bursting behavior. Sites representing frontal space near 0° azimuth were targeted. ITD tuning was determined via dichotic stimulation delivered through speakers (ED-21913-000, Knowles Electronics) positioned 5 mm from the tympanic membrane. Tungsten electrodes were removed and replaced with glass electrodes in the same location (1.5 mm borosilicate glass, 10–20 μm tip), containing 10% biotinylated dextran-conjugated tetramethylrhodamine 3000 MW (microruby, #D-7162; Thermo Fisher) or 10% lysine fixable dextran 3000 MW (Texas-red, #D3328; Thermo Fisher) in 1% potassium chloride solution. Chloridized silver wires were used to record physiologic responses to confirm injection location. Ionophoretic injections were performed using 7-s on/off cycles of 3 μA for 15 min. Glass electrodes were withdrawn and the procedure was repeated on the opposite tectal lobe. In 1/6 injections, the external nucleus of the inferior colliculus (ICX) was directly targeted (Table 1). Following injections, craniotomies were disinfected with 1% chloroptic ointment. After 3- to 7-d survival time, owls were anesthetized using 5% isofluorane + 1:1 nitrous oxide/oxygen. Heparin (300 U) was injected into the left cardiac ventricle and the owl was perfused with 0.1 M phosphate buffer (PO4, pH 7.4), followed by 4% paraformaldehyde in PO4 buffer (for STED imaging; see below for tissue preparation for SBEM imaging). Brains were removed and placed in 30% sucrose and 0.1 M PB for 24 h. Tectal lobes were isolated, sectioned at 50 μm on a vibratome (Leica), and mounted with an antifade agent (Prolong gold, P36930; Thermo Fisher).

**Table 1.** Owls

Adult owls (>250 d old)					
Owl	Side	Injection site	Tracer	Imaged cells	Imaging method
246	L	Deep OT	Microruby	1	SBEM
213	R	Deep OT	Microruby	6	STED
213	L	Deep OT	Microruby	8	STED
229	R	Deep OT	Microruby	8	STED
972	R	ICX	Microruby	11	STED
972	L	Deep OT	Texas Red	7	STED
				40	
Juvenile owls					
Owl	Purpose	Age (d)	Patched cells	Imaged cells	
2	Intrinsic prop.	35–40	4	3	
3	Intrinsic prop.	35–40	5	3	
4	Intrinsic prop.	35–40	5	7	
5	Intrinsic prop.	35–40	4	1	
6	Intrinsic prop.	35–40	2	2	
7	Synaptic stim.	40–50	2	0	
8	Synaptic stim.	40–50	1	0	
			23	16	

Owls and procedures.

### STED imaging and morphometric analysis

Imaging was performed on a Leica TCS SP8 STED 3× confocal microscope (Leica Microsystems), using a 100× oil-immersion objective (HC PL APO CS2, 1.4 NA; Leica Microsystems). Parameters for STED imaging were white light laser, line 551 nm for stimulating, and 660-nm depletion laser, both between 20% and 40%. Image frame size was 4800 × 4800. Step size for *z*-stacks was 17.44 μm, resulting in pixel/voxel dimensions of 0.024, 0.024, and 0.18 μm (*x*, *y*, and *z*, respectively). Huygens Professional SVI software was used for deconvolution of all images, using a theoretical point spread function (PSF). All well-labeled neurons in the rostrolateral aspects of IC were imaged (*n* = 39 cells in perfusion-fixed tissue from three adult owls; *n* = 16 cells in immersion-fixed tissue from five juvenile owls as described later).

STED images were analyzed using Imaris 8.1 (Bitplane AG). Surface creation was used to measure surface area and volume of the soma and primary dendrites. To isolate the primary dendrite, the slice tool was used to separate the base of the dendrite from the soma at the point where the dendritic caliber first became uniform (i.e., avoiding sharp curvature at the interface). The cross-sectional surface of the ensuing dendritic face was used to measure thickness of primary dendrites.

Cell classification was accomplished using Imaris’ 3D view. For adult tissue, cells containing toric spines were classified as Type I, while cells lacking toric spines were classified as Type II. For juvenile tissue the cell population was more heterogeneous. Cells were not classified as Type I or Type II but scored for the presence or absence of toric-like spines.

### SBEM

In one owl, microelectrode recordings were made as described above followed by multiple tracer injections in deep OT to increase the likelihood of generating a completely filled neuron. The owl was perfused as before but with 1% glutaraldehyde in cacodylate buffer (Wilke et al., 2013); 150-μm vibratome sections were collected and shipped to University of California San Diego for further processing and imaging. Sections were freeze-fractured, reacted en bloc with streptavidin-HRP (which binds to the biotin moiety on microruby) and detected using diaminobenzidine (DAB). Light microscope image (LM) of the section at this point was obtained. Following osmication and staining with heavy metals the tissue was extremely opaque to visible light, obscuring internal structure. To target the labeled cell for SBEM volume imaging, a Zeiss Versa 510 x-ray microscope (XRM) was used to generate 3D tomographic volumes relative to tissue landmarks with 0.7-μm pixel resolution. Based on the XRM volume, a 45 × 75 × 75 μm tissue block centered on the labeled cell was targeted and imaged with a Gatan 3View unit on a Zeiss Merlin SEM at 2.5 kV, with a magnification of 2800×, 15 × 15 k raster size, and 1-μs pixel dwell time. The final pixel size was 5 nm and the Z step size was 70 nm. The imaging parameters yield sufficient resolution to identify synaptic contacts while maximizing the rate of data collection. The SBEM image volume was postprocessed for proper alignment at University of California, San Diego and annotated at University of California, Davis.

### SBEM analysis

A team of eight annotators used the open source platform IMOD (https://bio3d.colorado.edu/imod/) to analyze the SBEM image volume. It contained 54 cell nuclei representing six distinct cell types (neuronal and glial somatic profiles), four putative space-specific neurons, one of which was well labeled throughout entire cytosolic compartment including spines, an estimated 100s of myelinated axons, 1000s of unmyelinated axons and 10,000s of synapses. The labeled space-specific neuron was traced through all sections, meshed and rendered in 3D. This reconstruction includes the entire soma and proximal dendrites but not distal dendrites as they extended outside of the volume (space-specific neuron dendritic fields can be ∼200 μm in diameter). Each toric spine was numbered and isolated for further analysis. The surface areas and contour volumes were determined using the *imodinfo* command, which calculates surface area by adding the areas of the triangles making up the mesh and the volume by summing the areas of each contour times the Z distance connecting the contours. The number of holes was assessed by visual inspection of the 3D model. Because the cytosolic label was dense, in most cases it was not clear whether these tori were cytoplasmically contiguous or interrupted by tight junctions, although one clear example of each was found.

Twenty-seven toric spines representing the full spectrum of complexities were selected for further analysis. All unlabeled axons innervating these 27 spines were traced outwards from their points of synaptic contact until the axonal process became ambiguous or reached the end of the volume.

### Synapse identification in SBEM

Synapses were identified by the presence and distribution of presynaptic vesicles. Active zones were identified using the following criteria: (1) a cluster of five or more presynaptic vesicles, (2) vesicle cluster apposed to the presynaptic membrane, (3) parallel presynaptic and postsynaptic membranes (no gaps), (4) these features present in at least two consecutive sections. In some cases, the postsynaptic density (PSD) could be discerned but the presence of the dark DAB label made this an unworkable requirement.

### Axonal trajectory analysis

Seven spines whose axons had been extended for longer distances without ambiguity were selected for trajectory analysis. The length, number of boutons and outgoing trajectories at each axonal end were measured. Length was measured by manual skeletonization. Boutons were defined as axonal swellings containing presynaptic vesicles. Trajectories were determined by marking the *xyz*-coordinates of the skeleton endpoint as well as a second internal skeletal point within 2 μm of the skeleton endpoint. A small number of axons branched and were split into segments. If the axon had a clear main segment with a smaller side branch it was classified as a “T” branch and the axon was split into two segments; the main segment and side branch. The length and number of boutons were measured separately for each segment. For trajectory analysis, only the two ends of the main segment of these axons were included. If the axon branched into two pieces that were comparable and a “main segment” could not be distinguished, it was classified as a “Y”-branch and split into three segments at the branch point. End 1 of each segment was defined as the open end, and end 2 was the branch point. Only one analyzed axon was classified as a Y-branch. For this axon, the trajectory was measured at all three open ends.

Axonal trajectories were analyzed by custom scripts written in MATLAB (MathWorks). The two points at each end were converted to a 3D vector with the outside point as the vector head and the inside point as the vector tail. All *z*-coordinates were scaled by a *z* ratio of 13.78 pixels in *x-y/z* section. Each end vector was scaled to a length of 1 and plotted in 3D with its tail at the origin. End vectors were grouped together by spine, with the two vectors from the same axon paired together. To determine whether input trajectories were clustered, the distance between vectors was measured as the minimum angle of arc drawn from the head of one vector to the head of the other. Vector distance (θ) was calculated from the dot product of the Euclidean vectors:

$$\cos\emptyset =\frac{\underset{u}{\to }.\underset{v}{\to }}{\underset{u}{\to }\text{*}\underset{v}{\to }}$$

Distances were calculated pairwise between each end vector and every other end vector on the same spine, except the other end of the same axon. The distance between the two end vectors from the same axon was called “angle of separation” to differentiate from other vector distances. This is a proxy measure for curvature of the axon. A perfectly straight axon yields an angle of separation of 180°. The single Y-branched axon on TS24 was included in the vector plots but excluded from the distance calculations for simplicity.

### Connection fraction (CF)

Potential connectivity for TS1 and TS7 was assessed by partial reconstruction of all unmyelinated axons that passed within 2 μm of the spine. CF was calculated as (the number of axons that synapse with the spine/the total number of axons within 2 μm). To determine the probability envelope of observing the actual distribution of active zones across each potential axonal input, a bootstrap analysis was constructed based on the simple assumption of equal access. The simulation was run 10,000 times for each spine to determine mean values and ±2 SD.

### *In vitro* slice preparation

Six juvenile owls (35–40 d old) were used for current clamp experiments to determine intrinsic properties and two juveniles (40–50 d old) were used for voltage-clamp experiments to measure EPSCs. Juvenile owls were anesthetized with isoflurane and perfused transcardially with heparin-containing (300 units/l) artificial CSF (aCSF; 124 mM NaCl, 1.3 mM KCl, 1 mM NaH_2_PO_4_, 26.2 mM NaHCO_3_, 1.3 mM MgCl_2_, 2.5 mM CaCl_2_, and 11 mM glucose). Brains were rapidly removed, blocked, and placed in ice-cold modified aCSF (248 mM sucrose, 11 mM glucose, 1.3 mM KCl, 1 mM NaH_2_PO_4_, 26.2 mM NaHCO_3_, 1.3 mM MgCl_2_, and 2.5 mM CaCl_2_). Horizontal midbrain sections (250 μM) containing ICX and lateral shell of the central nucleus of the inferior colliculus (ICCls) were cut on a vibratome (Leica VT1200S) and transferred to an incubation chamber containing aCSF at 32°C for 30 min before moving to aCSF at room temperature until used for recordings. Recordings were made in a submersion chamber perfused with aCSF (2 ml/min) at room temperature. All solutions were bubbled with 95% O_2_-5% CO_2_ continuously.

### Patch-clamp recordings

Whole-cell patch-clamp recordings were made from visually identified cells in the ICX region using borosilicate glass pipettes (2–4 MΩ). For current-clamp experiments, pipettes were filled with K-methanesulfonate-based internal solution (135 mM CH3KO3S, 10 mM HEPES, 1 mM EGTA, 4 mM Mg-ATP, 0.4 mM Na-GTP, and 10 mM phosphocreatine; 310 mOsm) containing 0.5% biocytin (Thermo Fisher). For voltage-clamp experiments, a Cs-methanesulfonate-based internal (120 mM CH_3_CsO_3_S, 15 mM CsCl, 8 mM NaCl, 10 mM TEA-Cl, 10 mM HEPES, 0.5 mM EGTA, 2 mM QX314, 4 mM Mg-ATP, and 0.3 mM Na-GTP; 310 mOsm) was used. All chemicals were from Sigma. Recordings were acquired using a Multiclamp 700B amplifier (Molecular Devices), digitized at 20 kHz with a Digidata 1550 digitizer (Molecular Devices), and low-pass filtered at 8 kHz. Evoked EPSCs were elicited in cells voltage-clamped at –70 mV in response to a brief electrical stimulus (0.2 ms, 20–50 μA) delivered through a concentric bipolar stimulating electrode (FHC). The stimulating electrode was placed locally in the ICCls. Synaptic blockers (bicuculine: 20 μM; NBQX: 10 μM; both from Sigma) were washed in during recordings, as indicated. Slices were immersion-fixed in 4% paraformaldehyde and stained with SA-488 for STED imaging.

### Data analysis

Resting membrane potential (in mV) was measured while injecting no current (*I* = 0) immediately after breaking into a cell (average of first 500 ms). Membrane capacitance was estimated using a single-exponential fit to the first 200 ms of the response to a 0.5-s injection of hyperpolarizing current. Membrane resistance was measured from the linear portion of a current clamp input-output curve as the slope of the line fitted to the voltages recorded at increasing current injections. Action potentials were elicited by increasing depolarizing steps (0.1–3 nA, 0.5 s) delivered every 15 s. Spikes were identified using a 20 mV/ms threshold of the first-order derivative of membrane potential. Results were identical when a more conservative 50 mV/ms detection threshold was applied. Spike onset was determined using the 2nd-order derivative (maximum acceleration) of membrane potential within a 2-ms time window before and after threshold crossing. Spike peak voltage (V_P_) was defined as the maximal V within a 2-ms time window from threshold crossing and was used to calculate spike amplitude (peak V minus V at spike onset). First spike latency was defined as the time between stimulus onset and first spike onset, and inter-spike interval as the time between consecutive spike onsets. All data were analyzed with custom scripts written in MATLAB.

## Results

**Figure 1.**
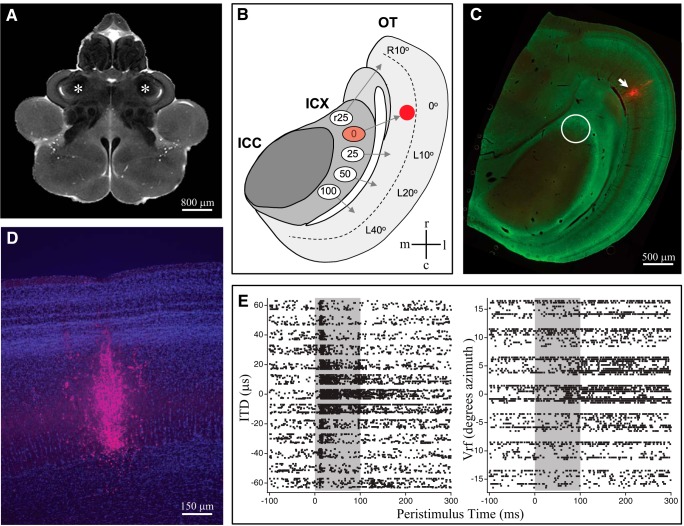
*In vivo* labeling of SSNs. ***A***, MRI image of owl brain with tectal lobes indicated by asterisks. ***B***, Diagram of horizontal section through R tectal lobe. ITD tuning of SSNs in ICX is indicated in microseconds left-ear leading. Visual receptive field (Vrf) location is indicated in degrees azimuth SSNs project to the deep layers of the OT where the auditory map aligns and integrates with visual space map indicated in degrees azimuth. Tracer injection at 0 μs/0° in the dOT (solid red) retrogradely labels SSNs at the cognate map locations in the inferior colliculus. ***C***, Representative horizontal section (50 μm thick) shows location of injection site (arrow) and location of retrogradely labeled neurons in the inferior colliculus (circle). Green label is immunostaining for CaMKII which is highly expressed in the lateral aspects of ICX. The border between the ICCls and ICX is not well delineated. ***D***, Higher magnification view of a representative injection (red) in the dOT. Blue label is DAPI staining. ***E***, Raster plots displaying the auditory (left) and visual (right) tuning recorded at the site of injection. IC, inferior colliculus; ICCls, lateral shell of the central nucleus of the inferior colliculus; ICX, external nucleus of the inferior colliculus; SSN, space-specific neuron.

The strategy for labeling space-specific neurons is shown in Figure 1. Tungsten and glass electrodes were used to record multiunit activity in the tectal lobes, which include the inferior colliculus (IC) and OT. The lobes are shown in a magnetic resonance image of an intact owl brain at mid-transverse plane (Fig. 1*A*). Space-specific neurons found in the ICX are tuned for distinct values of ITD, as illustrated in Figure 1*B*. Their auditory spatial receptive fields are constructed from convergent inputs which originate in the ICCls and other structures not shown (see Discussion). Space-specific neurons were labeled by injection of the tracer microruby or Texas-red in the dOT (5/6 injections), which receive monosynaptic input from space-specific neurons. Injection sites were confined to the deep layers with minimal rostrocaudal spread (Fig. 1*C*,*D*). ITD and visual spatial tuning at the injection sites (Fig. 1*E*) represented frontal space; as expected, the retrogradely labeled neurons were found within the region of the inferior colliculus representing frontal space, shown by the white circle in Figure 1*C*. This region includes the full mediolateral extent of ICX as well as lateral aspects of ICCls. Because no cytoarchitectonic marker that delineates the border between ICCls and ICX (Takahashi et al., 1987; Wagner et al., 2003) was compatible with the three tissue preparation methods used in this study (glutaraldehyde perfusion, 150-μm section; paraformaldehyde perfusion, 50-μm section; *in vitro* slice, 250-μm section), neurons were not assigned to one or the other structure. In one case the injection was directly targeted to ICX; labeled neurons resulting from this injection were indistinguishable in both location and morphology from those labeled by the five dOT injections and therefore included in the analysis below. No neurons outside of the ICCls-ICX microcircuit were analyzed.

**Figure 2.**
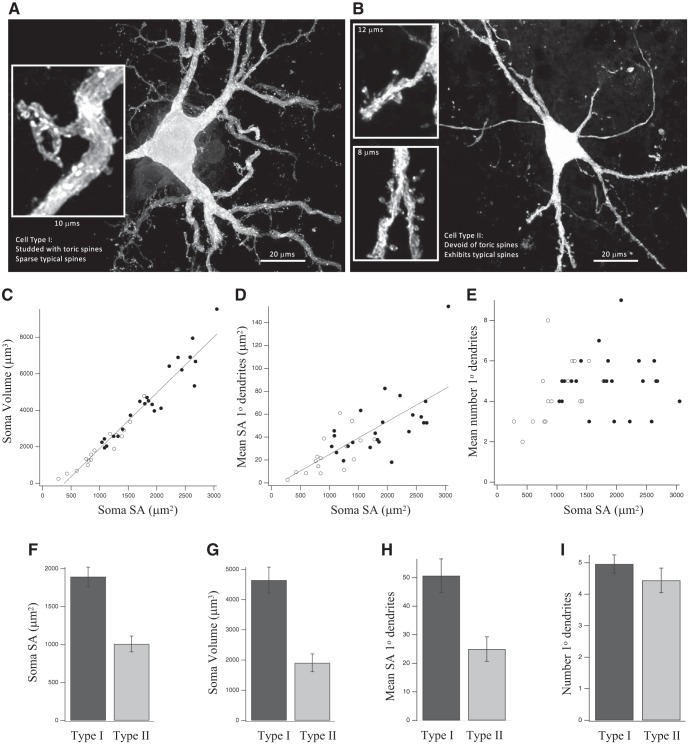
Type I and Type II SSNs. STED images of representative examples of Type I (***A***) and Type II (***B***) SSNs; *N* = 39 SSNs total, 23 Type I (solid circles in ***C–E***) and 16 Type II (open circles in ***C–E***). ***C***, Soma volume versus surface area across both types: *r* = 0.968, *r* ^2^ = 0.936, *p* < 0.00001. ***D***, Mean cross sectional surface area of primary dendrites versus surface area of the soma: *r* = 0.728, *r* ^2^ = 0.530, *p* < 0.00001. ***E***, Mean number of primary dendrites versus surface area of the soma: *r* = 0.243, *r* ^2^ = 0.059, *p* = 0.1356. ***F***, Mean somatic surface area (Type I = 1895 μm^2^ versus Type II = 1008 μm^2^; *p* < 0.00001). ***G***, Mean somatic volume (Type I = 4648 μm^3^ vs Type II = 1909 μm^3^; *p* = 0.00006). ***H***, Mean cross sectional surface area of primary dendrites (Type I = 50.7 μm^2^ vs Type II = 25.0 μm^2^; *p* = 0.00148). ***I***, Mean number of primary dendrites (Type I = 4.96; Type II = 4.44; *p* = 0.271). Comparisons for ***F–I*** were based on Mann–Whitney *U* test. Error bars are SEM.

The tracer often produced complete cell-fills, revealing fine aspects of dendritic structure, similar to previous reports using *in vivo* labeling (Peña and Konishi, 2001) or immunostaining for CaMKII (Rodriguez-Contreras et al., 2005; Niederleitner and Luksch, 2012). From these studies, it was known that space-specific neurons are large multipolar cells with dendritic protrusions which often included typical spines. Unlike previous studies, we used high-resolution methods to reveal, for the first time, their ultrastructure. STED microscopy of 39 well-labeled space-specific neurons derived from five injections in three owls revealed two cell classes within a spectrum of overlapping morphologic features. The most striking difference was the presence of highly atypical dendritic spines on a subset of cells, defined here as Type I space-specific neurons. These large structures exhibited tubular build, tended to lack spine heads and were often riddled with holes (Fig. 2*A*). The morphologic diversity of toric spines was so large that no single feature distinguishing them from typical spines was present in all. Type I space-specific neurons were studded with toric spines and a paucity of typical spines, sometimes none (Fig. 2*A*). Type II space-specific neurons, defined here, were studded with a high density of typical spines and devoid of toric spines (Fig. 2*B*). In addition, Type I space-specific neurons tended to have larger somas (Fig. 2*C*,*F*,*G*) and thicker primary dendrites (Fig. 2*D*,*H*). The number of primary dendrites was not significantly different (Fig. 2*E*,*I*).

**Figure 3.**
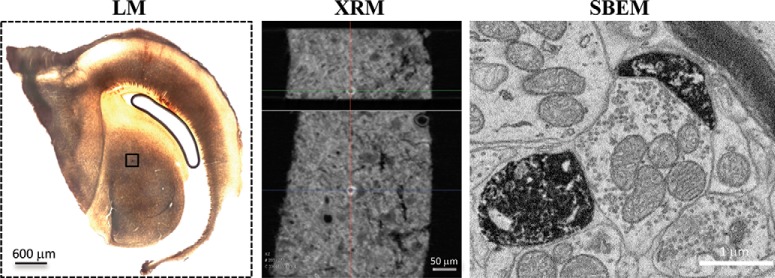
SBEM pipeline and SSN reconstruction. Light microscopy (LM) of 150-μm-thick section reacted en bloc for DAB, before osmication. The labeled SSN is shown in the box and appears near the border of ICCls and ICX. XRM, x-ray microscopy was used to locate the SSN after osmication; SBEM was used to image a volume 75 × 75 × 45 μm at 5-nm resolution.

While the resolution of STED was sufficient to identify toric spines when they presented in the right orientation, high anisotropy (∼40 nm lateral, ∼600 nm axial resolution) introduced unacceptable distortions to the apparent 3D structure of spines presenting in other orientations (the majority of spines). Combined with high morphologic diversity and narrow tubular structure, volumetric measurement (toric vs typical) were not informative for the population of spines as a whole. We therefore turned to SBEM. Space-specific neurons were labeled as before, this time with multiple injections in the deep OT to increase the chance of detection, the brain perfused with glutaraldehyde and reacted en bloc with DAB to produce a durable reaction product that survived all subsequent processing. One well-labeled space-specific neuron was found on the ICCls-ICX border (Fig. 3, LM panel). After osmication the labeled space-specific neuron was isolated using x-ray microscopy (Fig. 3, XRM panel) and a 75 × 75 × 45 μm volume (253,125 μm^3^) imaged at 5 nm lateral resolution.

**Figure 4.**
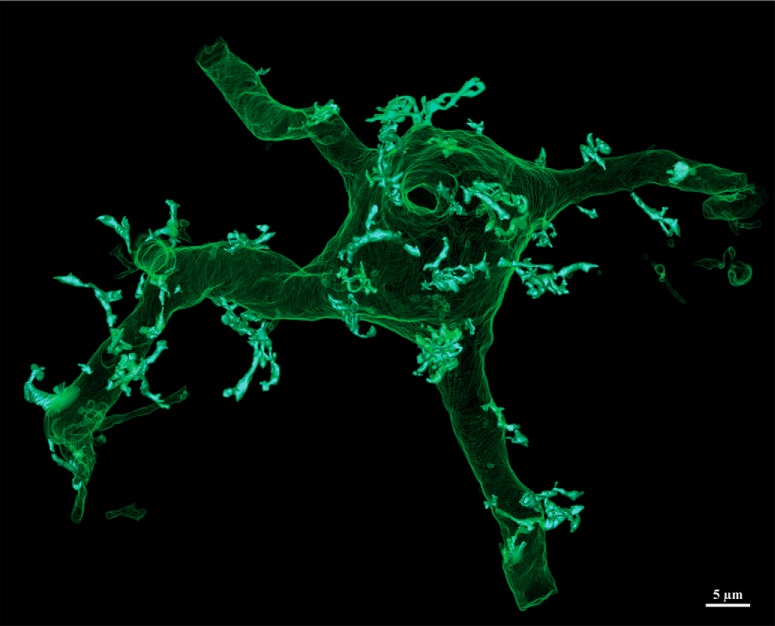
Reconstructed Type I SSN laden with toric spines. Full reconstruction of all labeled processes contained within the volume. Somatodendritic architecture is rendered in mesh green; toric spines in solid white.

**Figure 5.**
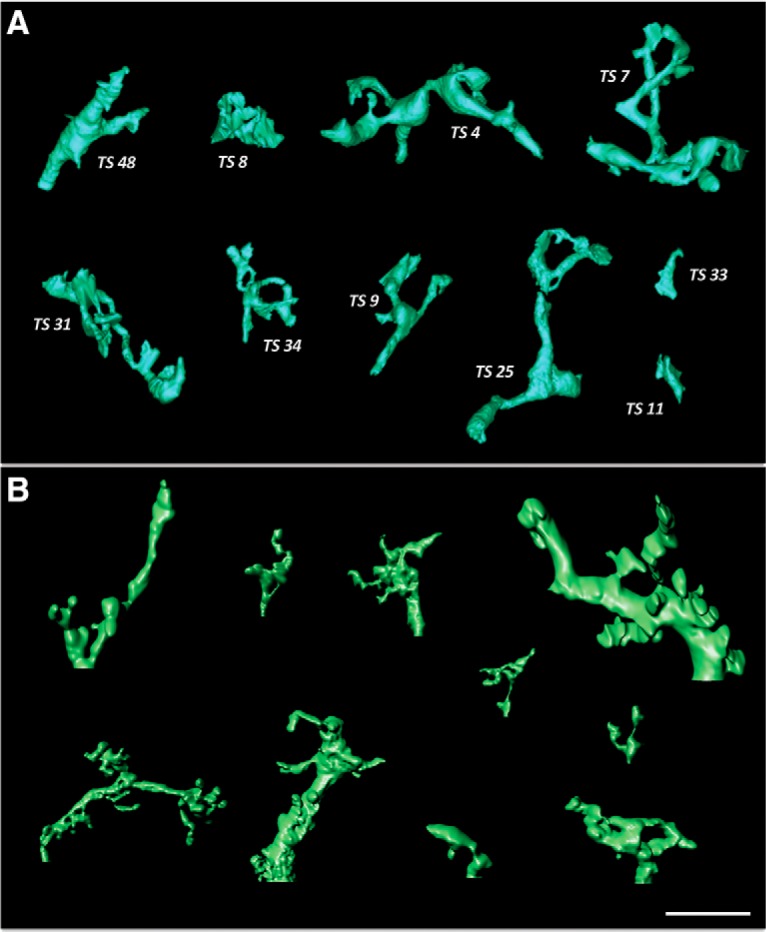
Toric spine zoo. ***A***, SBEM-based reconstructions of 10 example toric spines, selected to cover the range of diversity in size, shape and complexity. Holes were present in 37% of spines (29/78 spines). Note the relatively uniform process diameter and relative lack of spine heads. ***B***, STED-based reconstructions of 10 example toric spines, selected as above. The apparent ultrathin features result from thresholding a locally variable concentration of intracellular tracer as opposed to volumetric reconstruction based on plasma membrane tracing in SBEM. Otherwise, the population of spine morphologies derived from each imaging method appear indistinguishable. Scale bar = 5 μm (both panels).

The DAB reaction product completely filled the neuron, which received profuse synaptic contacts from many unlabeled afferents (Fig. 3, SBEM panel). Volumetric reconstruction revealed a large multipolar neuron (Fig. 4) resembling the Type I space-specific neurons identified by STED. In particular, the soma and its six thick primary dendrites were studded with 76 toric spines (TS1 – TS76) and devoid of typical spines. Comparison of representative “zoos” of toric spines identified by SBEM or STED (Fig. 5) indicated that these populations are qualitatively indistinguishable. A thin process resembling an axon emerged from near the base of one primary dendrite. No somatic axon was found.

**Figure 6.**
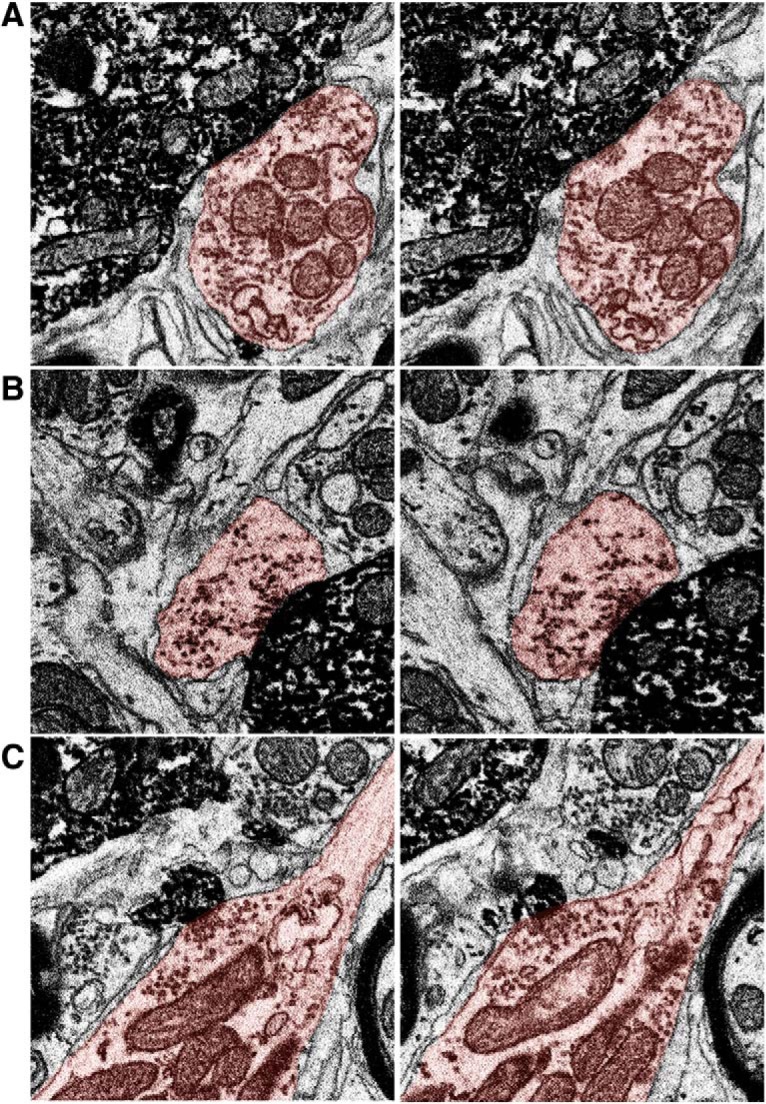
Synapse identification. Three representative synapses are shown onto the soma (***A***), dendrite (***B***), and toric spine (***C***) of the labeled SSN. For each synapse two successive EM sections are shown. The SSN is darkly labeled and the presynaptic compartment has been traced (light orange fill). The synapse on the soma is bifurcated, with two separate active zones from the same bouton. Bifurcated synapses were also common on the dendrites and toric spines. The axon shown in the bottom images makes synapses onto TS1 (larger spine) and TS62 (smaller spine; synapse shown in EM images), as well as the soma (data not shown).

**Figure 7.**
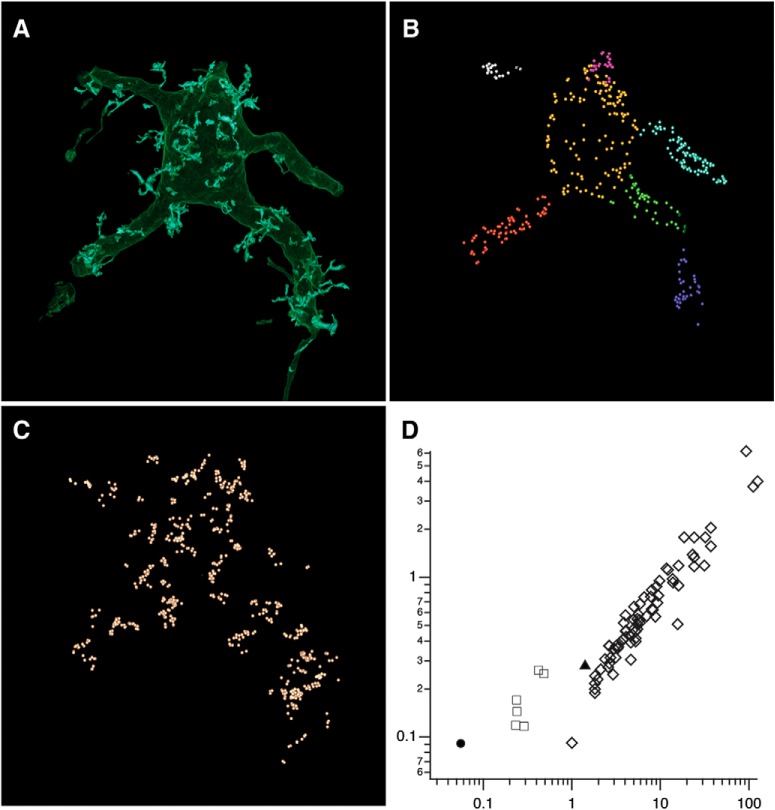
Distribution of synaptic input onto the labeled SSN. A team of annotators independently marked all active zones on the labeled SSN using criteria described in Materials and Methods. ***A***, Labeled SSN without active zones. Scale bar = 5 μm (***A–C***). ***B***, A total of 754 active zones was found on the soma (bright yellow; 228) and five primary dendrites (multiple colors; 526 total). ***C***, A total of 622 active zones were found on 76 toric spines (pale orange spheres). ***D***, Innervation density indicated by active zones per surface area (*y*-axis) or active zones per volume (*x*-axis) was on average largest for toric spines (open diamonds; mean = 0.81 and 12.6 for AZ/μm^2^ and AZ/μm^3^, respectively) than dendrites (open squares; mean = 0.177 and 0.32), soma (filled circle; 0.091 and 0.056), or thin process (filled triangle; 0.279 and 1.41).

To map synaptic input, all active zones were identified by a team of annotators using criteria defined in Materials and Methods. Active zones occurring on the soma, dendrites and toric spines appeared similar as a relatively homogenous population of chemical synapses with clusters of synaptic vesicles apposed to the postsynaptic membrane (Fig. 6). Bifurcated synapses with two or more active zones belonging to the same axonal bouton were occasionally observed on all compartments. 622 active zones were found on toric spines and 754 on the soma and dendrites, indicating that toric spines are a major input hub (Fig. 7*A–C*). The innervation density (# active zones per surface area) of toric spines was on average higher than that observed on the six primary dendrites, soma, or thin process (Fig. 7*D*).

**Figure 8.**
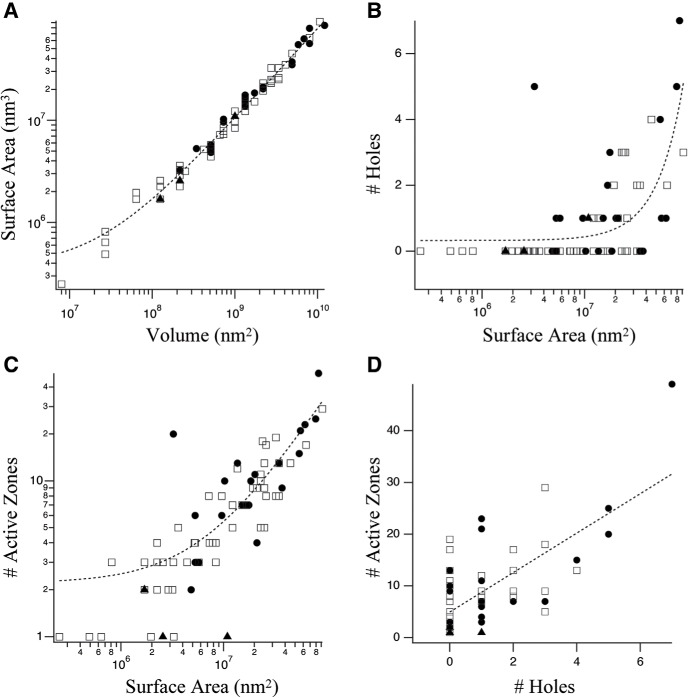
Morphometric analysis of the toric spine population. Volume, surface area, number of holes and number of active zones were quantified for each toric spine (*n* = 76) with 52 located on dendrites (open squares), 21 on the soma (filled circles), and 3 on the thin process (filled triangles). ***A***, Volume versus surface area. Data were marginally better fit (dashed line) by a power law (χ^2^ = 8.15 × 10^14^) than linear regression (χ^2^ = 9.28 × 10^14^; *r* ^2^ = 0.97, *p* < 0.00001. ***B***, Holes versus surface area: marginally better fit (dashed line) by power law (χ^2^ = 90.5) than linear regression (χ^2^ 91.1; *r* ^2^ = 0.42, *p* < 0.00001). ***C***, Active zones versus surface area: marginally better fit (dashed line) by linear regression (χ^2^ = 1254; *r* ^2^ = 0.73, *p* < 0.00001) than power law (χ^2^ = 1282). ***D***, Active zones versus holes: best fit by linear regression (*r* ^2^ = 0.50, *p* < 0.00001). There was a weak tendency for spines located on the soma to be larger than those located on dendrites, in both volume (mean values 3.13 vs 1.62 × 10^9^ nm^3^; *p* = 0.026 Mann–Whitney *U* test) and surface area (mean values 2.75 vs 1.50 × 10^7^ nm^2^; *p* = 0.016 Mann–Whitney *U* test). Spines on the thin process tended to be smaller though there were not enough data points to make a statistical comparison.

**Figure 9.**
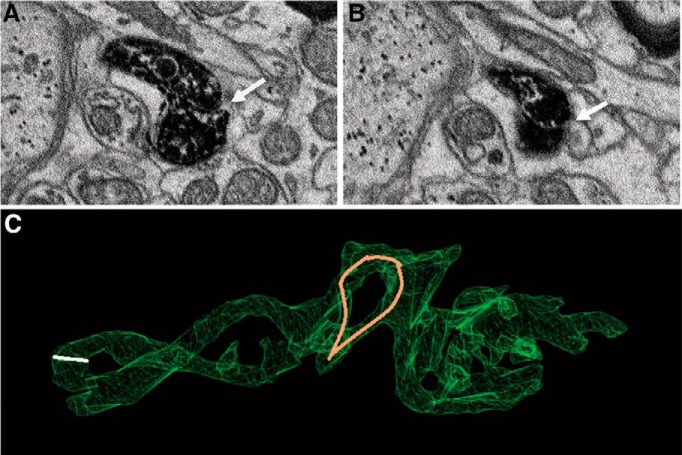
Examples of tight junction and cytoplasmic continuities. ***A***, ***B***, Consecutive EM sections showing a torus formed by a tight junction (white arrows). ***C***, Location of the tight junction shown above on TS1, indicated by white line. A separate torus exhibited cytoplasmic continuity across all sections (pale loop).

Quantification of the ultrastructural features of toric spines is shown in Figure 8. Volumes (mean = 1.99 × 10^9^ nm^3^) and surface areas (mean = 8.20 × 10^7^ nm^2^) varied over a wide range and were strongly correlated, the latter consistent with structures built largely of narrow tubes (Fig. 8*A*). Holes (up to seven on a single spine) were found on 37% of spines and were more prevalent in larger spines (Fig. 8*B*). Some holes were formed by tight junctions between two membrane-bound filopodial extensions while others appeared as cytoplasmic continuities (Fig. 9). The number of active zones per spine ranged from 1 to 49 and was strongly correlated with size (Fig. 8*C*) and number of holes (Fig. 8*D*). These data demonstrate large morphologic diversity within the toric spine population.

**Figure 10.**
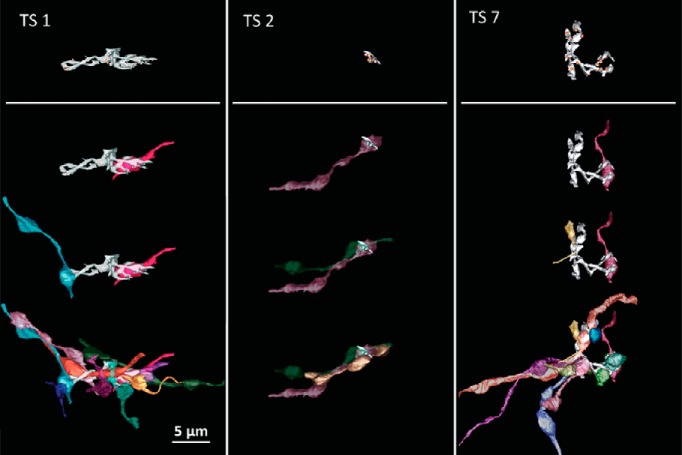
Toric spines receive multiple presynaptic inputs. Representative examples of axonal convergence onto three toric spines for TS1, TS2, and TS7. Top panels depict the spines (light gray) with all of its active zones (yellow circles). The lower panels show one, two and all axonal inputs (10, 3, and 10, respectively) added sequentially in color.

**Figure 11.**
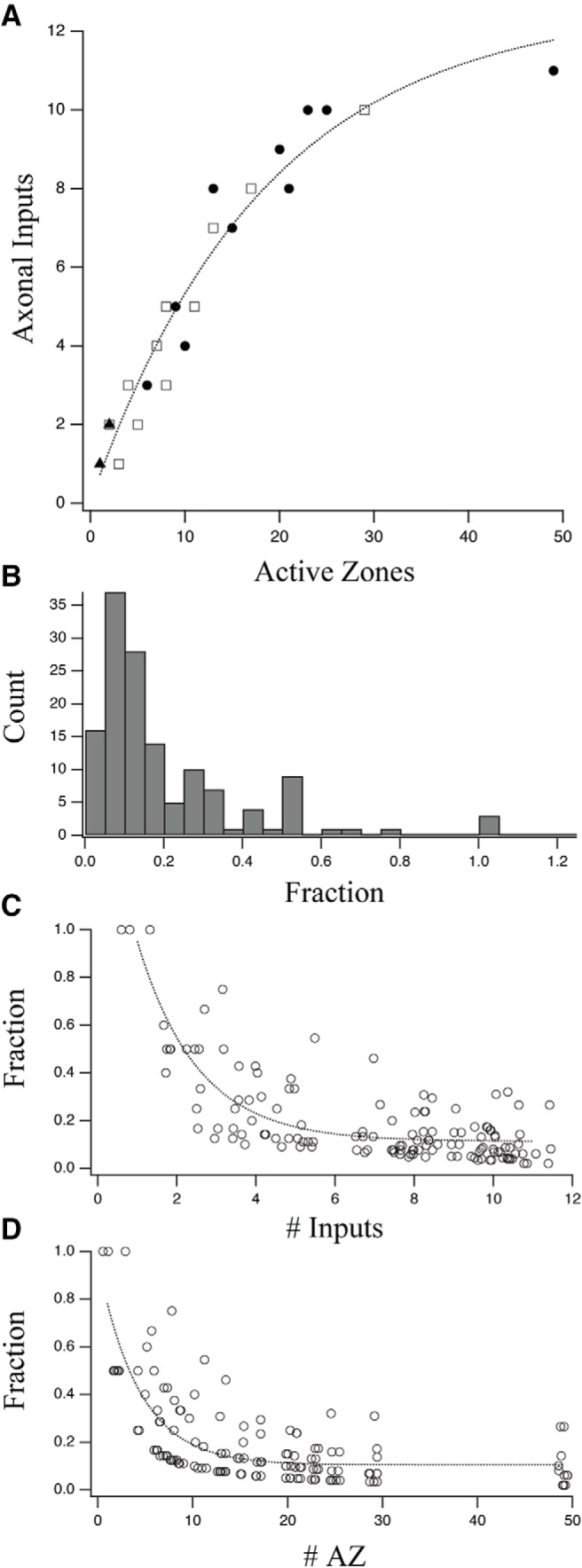
Synaptic convergence onto toric spines. Quantification of synaptic convergence for 27 representative toric spines: 13 located on dendrites (open squares), 11 on the soma (filled circles), and 3 on the thin process (filled triangles). ***A***, The number of axonal inputs strongly correlated with the number of active zones (linear regression: *r* ^2^ = 0.813, *p* ≤ 0.00001, χ^2^ = 49.4) and the data were better fit by an exponential function (χ^2^ = 14.59; dotted line). This was also true for both subpopulations of toric spines on the dendrites (linear regression: *r* ^2^ = 0.895, *p* ≤ 0.00001, χ^2^ = 8.88; exponential fit χ^2^ = 6.177) and on the soma (*r* = 0.837, *r* ^2^ = 0.700, *p* = 0.00125, χ^2^ = 25.46; exponential fit χ^2^ = 6.65). There was a weak tendency for spines located on the soma to have more inputs (mean values 7.1 vs 4.3; *p* = 0.03 Mann–Whitney *U* test) and active zones (17.9 vs 8.9; *p* = 0.026 Mann–Whitney *U* test) than those on the dendrites. ***B***, Histogram of the fraction of total synaptic input to each spine (see Materials and Methods) contributed by each axonal input (*n* = 138). ***C***, Input fraction decreased exponentially (dotted line) as the number of inputs increased. ***D***, Input fraction decreased exponentially (dotted line) as the number of active zones increased. For both ***C***, ***D***, the *x*-coordinate was randomly shuffled by ±0.5 for display purposes only.

To determine patterns of synaptic convergence, 27 spines representing the morphologic spectrum were selected for reconstruction of axonal inputs. Three examples are shown in Figure 10. In each case, the active zones derived from multiple axons: 10 axons provided 25 AZs to TS1; three axons provided six AZ to TS2; and 10 axons provided 23 AZ to TS7. In fact, the majority of toric spines (all except three of the 27 representative spines) received more than one axonal input. The number of axonal inputs strongly correlated with the number of AZs, and this was true for spines located on the soma or dendrites; spines on the soma tended to receive more inputs than those on the dendrites or thin process (Fig. 11*A*), consistent with their tendency to be larger.

To determine the degree to which inputs to a single spine were balanced or unbalanced in anatomical strength we measured the input fraction, defined as the number of active zones made by a single input divided by the total number of active zones received (metric range = 0–1). A low fraction indicates the input has a weak contribution to the overall innervation of the spine, while a high fraction indicates the input is a dominant driver. The distribution of input fractions was skewed low (mean = 0.20, SD = 0.19, *n* = 138) indicating the most common arrangement was integration of many individually weak inputs, a situation of anatomical balance (Fig. 11*B*). Large input fractions were mostly limited to spines that received few inputs (Fig. 11*C*) and active zones (Fig. 11*D*), both of which set a high lower limit on the possible values of input fraction. Overall, the common motif was anatomically balanced integration.

**Figure 12.**
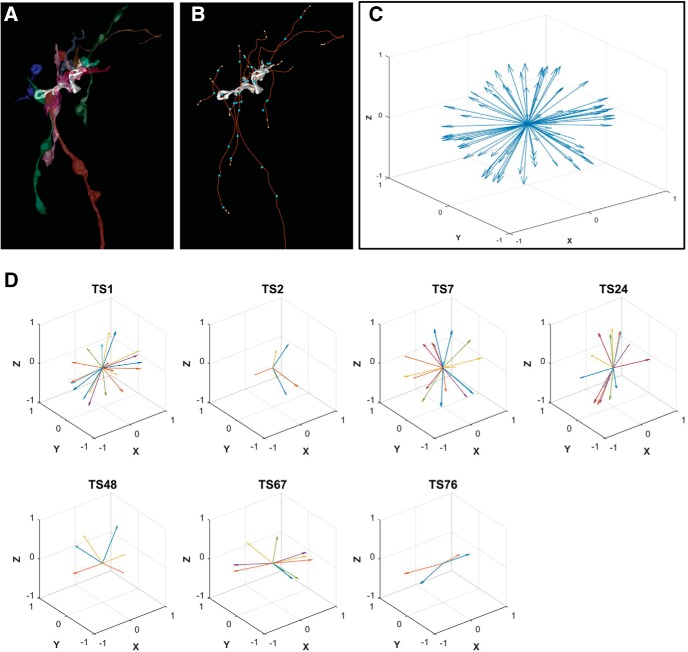
Trajectory analysis of axonal inputs. ***A***, Representative example trajectory analysis. Axons providing input (in this case to TS1) were extended to the edge of the volume or until a reconstruction ambiguity was encountered. ***B***, For a total of 40 axons synapsing onto seven toric spines, the length (red line), number of boutons (blue dots), and the outgoing trajectories at each end of the axon (small yellow dots) were measured (see Materials and Methods). ***C***, Axonal trajectories were represented as vectors through the two end points, scaled to a length of one and plotted in three dimensions with their tails at the origin (*n* = 81). ***D***, In plots 1–7, the vectors are grouped by spine and the ends of the same axon are paired visually by color within each plot.

**Figure 13.**
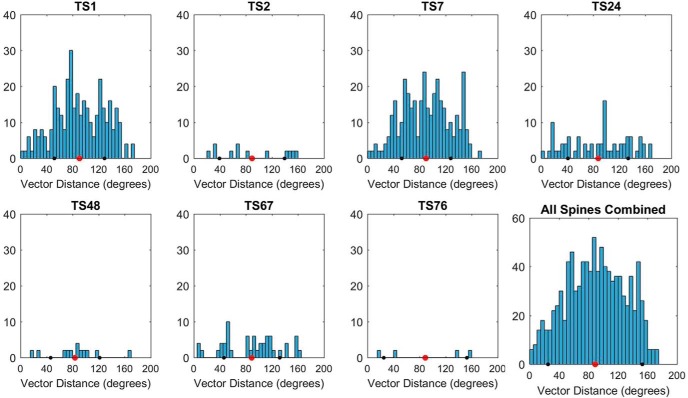
Angular distances between the input trajectories onto toric spines. Histograms of the angular distance between each trajectory vector and every other vector for each spine, excluding the vector from the other end of same axon. Distances were calculated with the dot product (see Materials and Methods). Mean across all spines = 89.7°; SD = 40; *N* = 78 vectors, 976 pairings; bin width = 5). A population of random trajectories predict a Gaussian distribution of distances with a mean of 90°, as observed from 6/7 spines and the overall population, while a population of bundled trajectories predicts a bimodal distribution with a mean of 90°, as observed for TS76.

**Figure 14.**
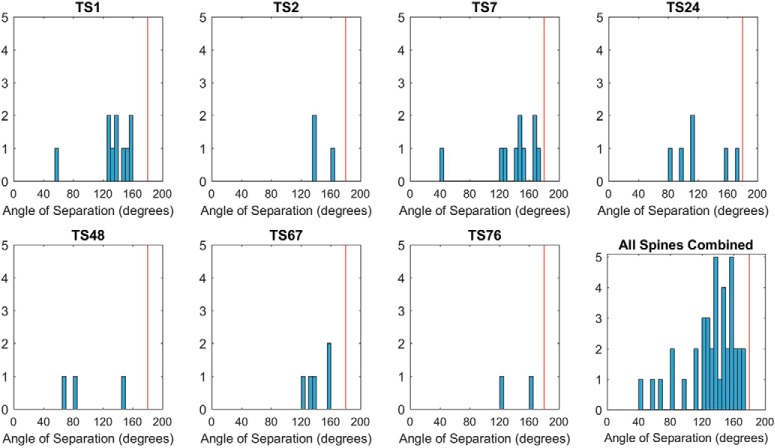
Angle of separation between paired ends. Histograms of the angular distance between the paired ends of each axon. Mean = 132.9°; SD = 31.5; *N* = 40. Linear trajectories predict a clustered distribution near 180°, as observed for the overall population, while highly curved trajectories predict a Gaussian distribution with a mean near 90°.

To determine whether multiple inputs derived from the same parent axon, seven spines whose axons had been extended for considerable distance without ambiguity were selected for trajectory analysis (mean length = 20.6 μm; see Materials and Methods; Fig. 12*A*,*B*). None of these 40 axons joined together. Further, the population of trajectories was distributed near randomly in 3D (Fig. 12*C*), and this divergent profile (Figs. 12*D*, 13) was evident at individual spines (with one exception) including all of the most complex spines. The axonal segments tended to exhibit minimal curvature between incoming and outgoing ends (Fig. 14). In total, wide divergence of axons with linear trajectories strongly suggests that the axons originate from diverse input sources (see Discussion).

**Figure 15.**
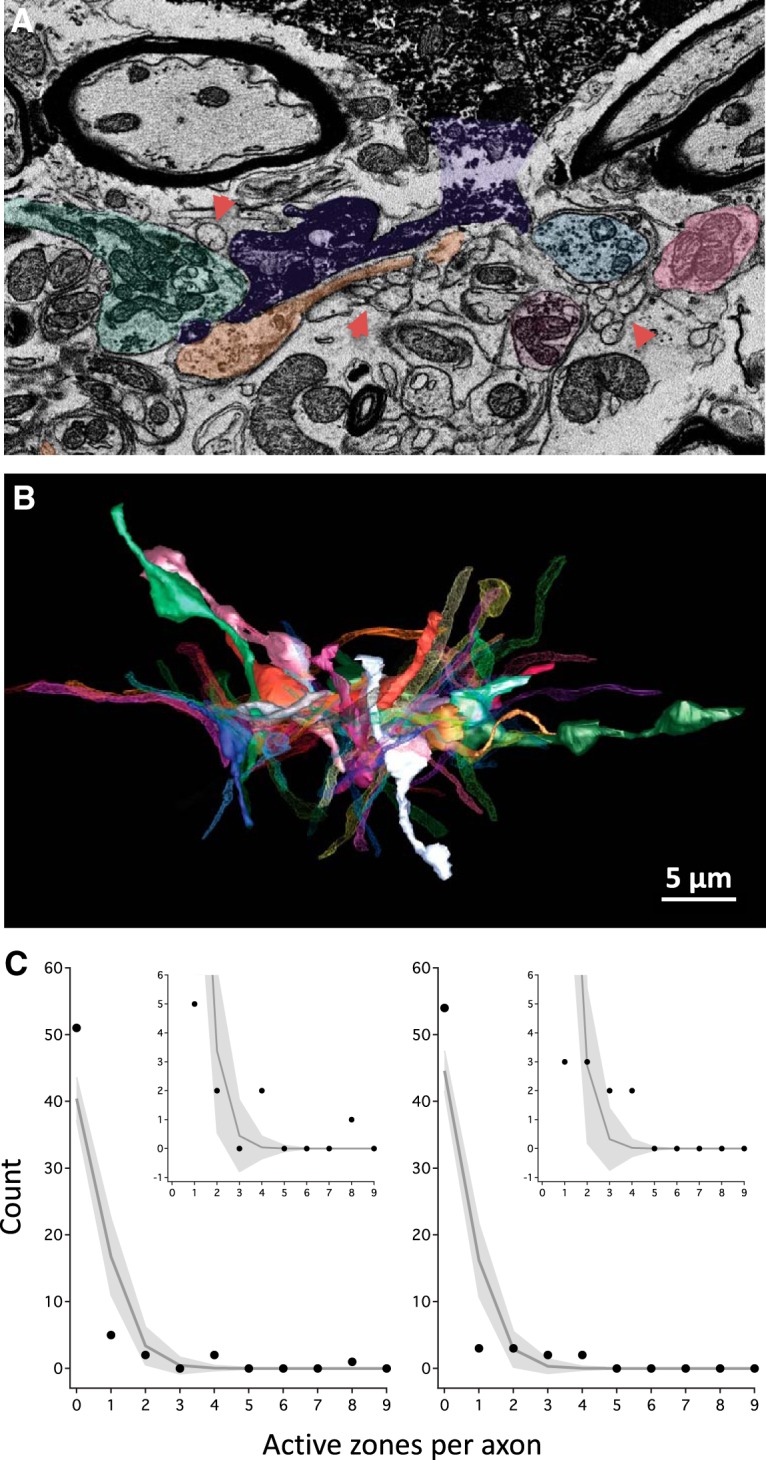
Potential connectivity of toric spines. To assess potential connectivity of TS1 and TS7, all unmyelinated axons that passed within 2 μm of the spine were reconstructed. ***A***, SBEM section showing four annotated axons (shaded in color) synapsing onto TS1 (dark purple). Arrows point to nearby axons which do not make any synapses onto TS1. ***B***, All 61 unmyelinated axons within 2 μm of TS1 (solid gray). There were also five myelinated long-range axons that came within 2 μm of TS1 (data not shown and excluded from this analysis; see text for explanation). In all, 10 unmyelinated axons synapsed onto TS1 (solid axons) and 51 did not (meshed axons). Thus, the CF for TS1 is 0.164. The CF of TS7 (data not shown) is 0.156 (10 connected axons, 54 unconnected axons). ***C***, Bootstrap analysis of potential connectivity of TS1 (left panel) and TS7 (right panel). Histograms indicate the relative frequency of observing axons connected with the specified number of active zones to TS1 in the real data (solid circles) and 10,000 simulations (gray line; light gray envelope indicates ±2 SD). In both spines, four of the 10 data points lie outside the envelope of expectation based on random connectivity. Insets display the same data over a magnified range.

To investigate the potential for toric spines to form synapses with novel inputs we investigated the CF of two complex spines, TS1 and TS7. Prior work defined “filling fraction” as the number of synapses formed on a postsynaptic target (in that case, a dendrite or neuron) divided by the total number of synapses available within a local volume of surrounding neuropil extending 2 μm in all directions away from the postsynaptic target (Stepanyants et al., 2002). This is the typical range of filopodial extensions that occur across circuits and species in healthy tissue undergoing experience-dependent synaptic plasticity (Grutzendler et al., 2002; Trachtenberg et al., 2002). We define a closely related concept, CF, which is the number of axons connected to the spine divided by the total number of axons that could be connected by virtue of proximity and lack of intervening myelin (Mishchenko et al., 2010). To determine CF, all unmyelinated axons within two μm of the target spine were reconstructed (Fig. 15*A*). Infiltrating TS1 were 61 unmyelinated axons, 10 of which were synaptically connected to it (CF = 0.164). For TS7 the numbers were 64 axons, 10 connected, CF = 0.156. These low CFs are on par with prior reports of filling fractions for circuits in mammalian tissue, e.g., 0.18 and 0.19 for two densely reconstructed hippocampal dendrites (Mishchenko et al., 2010).

Synaptic connectivity of the infiltrating axons could in principle be random or structured. To test this, we constructed a bootstrap analysis based on the assumption that each axon has equal ability to form an active zone with the target spine during a hypothesized epoch of microstructural plasticity. We determined the probability envelope for distributing the total number of AZs received (25 for TS1, 23 for TS7) across each potential axonal input (61 for TS1, 64 for TS7). The actual data fell well outside the probability envelope based on random access (Fig. 15*C*). This indicates that connectivity is structured such that certain axons have, or develop, preferential access. Such non-random connectivity is also found in mammalian cortex (Kasthuri et al., 2015) and thalamus (Morgan et al., 2016), suggesting a common principle for circuit development.

**Figure 16.**
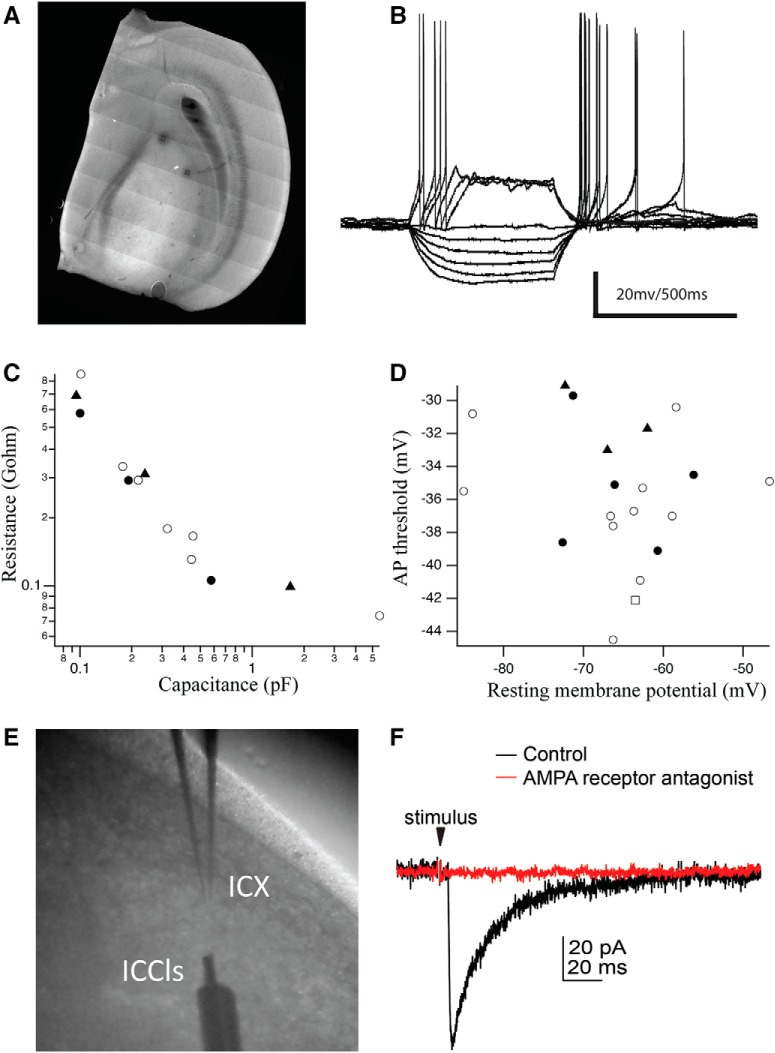
Patch clamp recordings. ***A***, Mosaic image of a 300-μm-thick acute horizontal section through the tectal lobe. Photobleached squares reveal locations of recorded neurons in ICX. ***B*,** Example of current step protocol used to measure intrinsic membrane properties (*n* = 20 cells, five slices, six owlets age 35–40 d). ***C***, Resistance versus capacitance for 13 cells. ***D***, Action potential (AP) threshold versus resting membrane potential for 20 cells. For both ***C*** and ***D***, filled circles = cells with toric, atypical, and typical spines (*n* = 5); filled triangles = cells with atypical and typical spines but not toric spines (*n* = 3); open squares = cells with no spines (*n* = 1); open circles = not determined (*n* = 11). ***E***, Electrical stimulation experiment with glass recording electrode in ICX and bipolar stimulating electrode in ICCls. ***F***, Example of electrically-evoked EPSC (*n* = 2) that was completely abolished by bath application of AMPA receptor antagonist NBQX.

**Figure 17.**
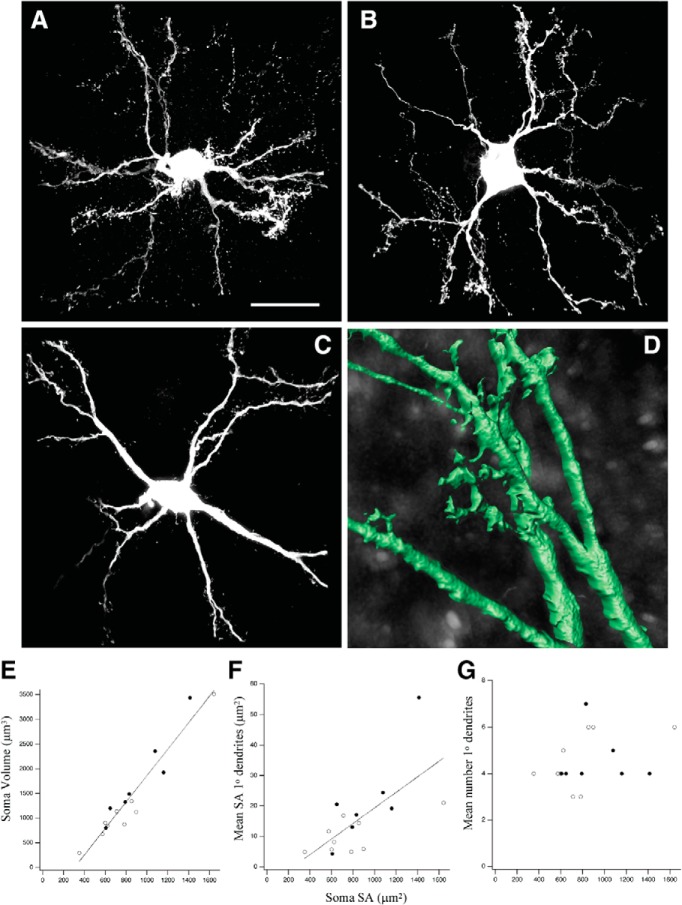
Morphologies of putative SSNs in juvenile owls. ***A–C***, Examples of the morphologic diversity of ICX cells in juvenile owls. Cells studded with a variety of spine types, atypical, branched, toric-like, and typical, were common and resembled Type I adult SSNs in multipolar structure (***A***, ***B***). A minority of juvenile cells exhibited relatively few spines (***C***). Toric-like structures with incompletely closed holes (***D***) were found on 7/16 juvenile cells (filled circles in ***E–G***). Scale bar = 30 μm (***A–C***) and 5 μm (***D***). ***E***, Soma volume versus surface area across both types: *r* = 0.962, *r* ^2^ = 0.925, *p* < 0.00001. ***F***, Mean cross sectional surface area of primary dendrites versus surface area of the soma: *r* = 0.676, *r* ^2^ = 0.457, *p* = 0.004. ***G***, Mean number of primary dendrites versus surface area of the soma: *r* = 0.323, *r* ^2^ = 0.104, *p* = 0.222.

To open a path for linking microanatomical patterns of synaptic convergence with functional measurement of neuronal computation, we developed an *ex vivo* slice preparation to record from space-specific neurons. Recording electrodes were targeted to large somata in ICX under visual guidance (Fig. 16*A*) and used to record membrane potential responses using a current-clamp protocol (Fig. 16*B*). Neurons (*n* = 20 from five owlets) exhibited a spectrum of intrinsic membrane properties (Fig. 16*C*,*D*; Table 2). Some neurons (*n* = 16) were filled with biocytin for retrospective imaging to determine cell type. In this population of neurons in juvenile owls, large atypical spines were prevalent including toric-like structures of narrow tubular build with multiple arms whose ends often infolded on themselves, but which infrequently presented as closed holes (Fig. 17*D*). The morphologic diversity was greater than in the adult space-specific neuron population, with many juvenile cells expressing some mixture of atypical and typical spines (Fig. 17*A*,*B*), while a minority (*n* = 5) lacked a high density of spines (Fig. 14*C*). Therefore, juvenile cells were not classified as Type I or Type II, but instead scored for the presence (*n* = 7) or absence (*n* = 9) of toric-like spines. There was a trend for juvenile cells with toric-like spines to have larger soma and thicker primary dendrites than those lacking them (Fig. 17*E–G*), although these differences were not statistically significant, and both juvenile and adult cells had on average the same number of primary dendrites (mean = 4.5 for both), a comparative profile similar to the differences between Type I and Type II adult cells (Fig. 2). In total, the juvenile cells appeared as developmental precursors to toric space-specific neurons. Finally, in separate voltage-clamp experiments, electrical stimulation of the known input source to ICX, the ICCls, evoked typical AMPA-mediated EPSCs (Fig. 16*E*,*F*; *n* = 2), consistent with all prior studies of this monosynaptic connection.

**Table 2.** Patch-clamp recordings

Cell	RMP (mV)	Resistance (GΩ)	Time constant (s)	Capacitance (nF)	Threshold (mV)	Peak (mV)	Threshold to peak (mV)	Spike width (ms)	Latency (ms)
**M203a**	–60.7	0.292	0.055	0.191	–39.1	18.8	57.9	0.6	5.4
**M203b**	–66.3	0.179	0.058	0.321	–44.5	18.4	62.9	0.5	6.2
**M203c**	–63.5	0.058	—	—	–42.1	10.6	52.7	0.4	70.9
**M203e**	–72.6	0.578	0.058	0.1	–38.6	28.6	67.2	0.8	12.3
**M204d**	–66.6	0.859	0.087	0.101	–37	32	69	1.2	7.7
**M204e**	–67	0.69	0.065	0.095	–33	26.4	59.4	1.3	4.1
**M204f**	–56.2	—	0.075	0.085	–34.5	33	67.5	1.2	10.4
**M204h**	–62.9	0.293	0.064	0.218	–40.9	36.6	77.5	0.5	30.3
**M204i**	–62.6	—	0.059	0.152	–35.3	31.1	66.4	0.5	65.4
**M205h**	–62	0.311	0.074	0.238	–31.7	29.2	60.9	1.1	63.3
**M205i**	–66.1	—	—	—	–35.1	35	70.1	0.8	31.2
**M205j**	–71.3	0.106	0.061	0.577	–29.7	40.6	70.3	0.8	28.3
**M205k**	–63.7	—	0.061	0.078	–36.7	43.1	79.8	0.9	20.1
**M205l**	–58.4	0.336	0.059	0.177	–30.4	27.4	57.8	0.5	78.4
**M206a**	–83.9	0.166	0.068	0.453	–30.8	26.6	57.4	1.1	24.6
**M206b**	–58.9	—	0.057	0.129	–37	16.6	53.6	1	36.9
**M206c**	–72.3	0.099	0.164	1.666	–29.1	34.1	63.1	1.1	43.3
**M206e**	–85	0.131	0.058	0.444	–35.5	36.2	71.7	0.9	27.3
**M207e**	–66.3	—	0.073	0.09	–37.6	29.6	67.2	1	7
**M207f**	–46.7	0.074	0.266	5.494	–34.9	22.1	56.9	0.8	6.3

Intrinsic membrane properties recorded from 20 cells across five juvenile owls age 35–40 d. All reported data are from cells that passed multiple quality controls.

## Discussion

We describe the morphology and synaptic innervation patterns of a novel brain structure, toric spines. Across circuits and species, toric spine morphology appears unique. In contrast, while their high integrative capacity is unparalleled in degree, it is evocative of reports of complex spine types found in mammalian thalamus, hippocampus and cerebellum. This suggests that toric spines may represent a standout example of a processing motif common across the animal kingdom. The discussion below critically analyzes toric spine morphology, integrative capacity, implications for cellular computation, potential role in plasticity, and concludes by outlining testable hypotheses to probe more deeply into their structure and function.

Both STED and SBEM imaging revealed a broad spectrum of spine morphologies. Features that varied markedly across the population include size, number of holes and degree of branching. Features that tend to be shared include narrow tubular build, paucity of bulbous heads, and wide necks at the process interface. This profile distinguishes toric spines from all previously documented spine types including typical thin, stubby, mushroom (Bourne and Harris, 2007; Chirillo et al., 2019) or branched spines (Bourne and Harris, 2008), and atypical dendritic structures including thorny excrescences, dendritic appendages or glomerular excrescences (Jones and Powell, 1969; Spacek and Lieberman, 1974; Rapisardi and Miles, 1984; Erişir et al., 1997; Morgan et al., 2016). Perhaps most striking, toric spines received up to 49 active zones contributed by up to 11 axonal inputs - a stark contrast to typical spines (e.g. Arellano et al., 2007) that compartmentalize single inputs or thorny excrescences which detonate their postsynaptic target (Table 3). Given their size and integrative capacity (discussed below), toric spines could be classified as atypical dendrites. We call them spines because, like typical spines, they are substantially smaller in caliber than the parent process, a size differential that exceeds the difference between higher and lower order dendritic branches.

**Table 3.** Comparison of spine types

	Typical mushroom	Thorny Excrescence	Toric
Size	Small	Large	Large
Neck	Thin	Thin	Wide
Spine head	Large	Large	Rarely apparent
Branched?	Rarely	Profusely	Sometimes
Holes?	No	No	Yes
Cellular location	Dendrites	Base of apical dendrite	Soma and dendrites
Synapses per spine	∼1	10+	10+
Axonal inputs	∼1	1–2	Up to 11
Function	Compartmentalization	Detonation	Integration

The morphologies, dimensions, patterns of synaptic convergence, and known or hypothesized functions are shown for typical mushroom spines (e.g. Arellano et al., 2007, their Fig. 2) thorny excrescences (e.g. Wilke et al., 2013, their Fig. 5) and toric spines.

The narrow tubular build of toric spines should produce a short length constant due to high axial resistance, and therefore predicts that single synaptic inputs made onto one spine are isolated from those onto another (Rall, 1974). At the same time neighboring inputs onto the same spine should be subject to summation. Compartmental simulations of the reconstructed space-specific neuron to test this intuition have so far been unsuccessful due computational limitations of available modeling platforms. In comparison, simulations of neocortical pyramidal cells reconstructed from light microscopy (i.e., lacking ultrastructural detail and therefore amenable to compartmental simulation) have shown that neuronal computation can be represented by a two-stage model in which individual dendritic branches perform the first-stage processing of summation of EPSPs (Poirazi et al., 2003). Active properties (voltage-gated ion channels) modify the rules of dendritic integration providing a range of operations from supra-linear to sub-linear. The second stage is linear summation of dendritic branch output at the soma. The multipolar structure of space-specific neurons is well suited to emulate this processing scheme with the significant addition of an earlier-stage operation at toric spines.

Direct tests of these computational strategies require whole-cell recording in combination with targeted synaptic stimulation. We demonstrate, for the first time to our knowledge, the ability to make high-quality patch-clamp recordings from ICX neurons. Development of this *ex vivo* preparation opens a path for understanding the casual connections between microanatomical convergence and cellular computation.

All large toric spines with analyzed convergence patterns (*n* = 24) were found to receive synaptic input from multiple axons, indicating that they are structured to act as integrators. Synaptic convergence onto branched spines and other atypical dendritic protrusions has been suggested, though not firmly established, by earlier studies. Pioneering work using serial EM to reconstruct rat cerebellar Purkinje cells demonstrated a complex spine type with up to five branches. Independent axonal boutons could be found on distal branches of the same spine, several microns apart, raising the possibility that branched spines receive inputs from multiple axons, though this remains untested (Harris and Stevens, 1988). The prevalence of branched spines on Purkinje cells has been estimated between 2% and ∼6% (4/64; Harris and Stevens, 1988; Lee et al., 2004) and ∼15% on adult-generated granule cells in the dentate gyrus, where they are also polyinnervated (Bosch et al., 2015), suggesting an important role in neuronal computation. Synaptic glomeruli in mammalian thalamus receive convergent input from anatomically and neurochemically diverse sources (Jones and Powell, 1969; Spacek and Lieberman, 1974; Erişir et al., 1997). In a recent connectomics study, two densely reconstructed glomeruli received input from 15 different thalamocortical afferents (Morgan et al., 2016), a level of convergence similar to that observed in our dataset. The central postsynaptic compartment of glomeruli are dendritic shafts from which multiple tiny excrescences emerge, distinct electrotonically from toric spines. Nonetheless, the above results in combination with our findings raise the intriguing possibility that synaptic convergence onto complex spine types is more common than currently appreciated.

The conclusion that toric spines are innervated by multiple input sources is supported by analysis of axonal convergence onto 27 spines and trajectory analysis for seven. Not one shared branchpoint was found (40 axons, mean length = 20.6 μm). Until the axons are traced back to their parent cell bodies, a single source explanation cannot be logically eliminated. We think it is highly unlikely because it would require parent axons to branch, diverge at least 10-100s of micrometer, then re-converge and terminate within an extremely small volume of tissue. There is no evidence for such an arrangement. Owls are known for unusual axonal specializations, delay lines (Carr and Konishi, 1988), yet these are found in the projection from nucleus magnocellularis to nucleus laminaris (Carr and Konishi, 1990), distant brainstem structures, not ICX. In fact, ICCls axons, which provide the bulk of input to ICX, exhibit typical terminal fields in which axonal branches diverge, not converge (DeBello et al., 2001; Rodriguez-Contreras et al., 2005; McBride et al., 2008; McBride and DeBello, 2015). In principle, short branchlets with terminal boutons could be within range of a toric spine, but because those branchpoints are mere microns away from the parent axon, such arbors could not have contributed to the divergent trajectories of much longer axonal segments observed in our dataset.

The fact that individual ICCls axons cannot account for the convergence results does not rule out ICCls axons as a potential input source to toric spines. In fact, space-specific neurons are known to receive input from multiple ICCls neurons encoding different sound frequencies. Integration across frequency is required to eliminate phase ambiguity and ultimately provide the owl with an accurate estimation of sound source azimuth. At the level of spiking output, frequency convergence is a non-linear operation. Thus, one hypothesis is that axons originating from multiple ICCls neurons encoding the same ITD but different frequencies converge onto individual toric spines where their EPSPs are combined and thresholded (Peña and Konishi, 2000, 2002). These predictions are testable using dual anterograde injections placed at different dorsoventral laminae, and whole-cell recordings, respectively.

A second possibility is that toric spines integrate ITD and ILD, a multiplicative operation. Binaural integration first occurs in the ICCls and is largely inherited by space-specific neurons in ICX. Yet the precise anatomical and functional boundaries between ICCls and ICX are unclear. Several cytoarchitectonic markers delineate the lateral border of ICX, and separately, the border of the core of the central nucleus. But on close inspection of commonly used markers calbindin, calretinin and CaMKII, the ICCls-ICX border appears as a gradient. Microelectrode surveys also support the idea of functional heterogeneity along this border. Moreover, while cytoarchitectonic markers were not routinely employed in our study (see Materials and Methods), estimates based on comparison to similar sections from other owls indicate that at least some Type I space-specific neurons, including the one in the SBEM volume, were located in ICCls. This is a minor revision to the notion that IC-OT projection neurons are strictly confined to ICX. More importantly, it motivates the second hypothesis, that toric spines in ICCls receive convergent input encoding behaviorally relevant ITD-ILD pairs, from contralateral ICC (for ITD) and contralateral dorsal lateral lemniscal nucleus pars posterior (for ILD), and perform a multiplicative operation (Peña and Konishi, 2001, 2004) via a different complement of ion channels. A third possibility is that both hypotheses are true: toric spines are multipurpose integrators whose computation can be customized to cellular need.

Other potential sources of input to toric spines include the arcopallial gaze field (Cohen et al., 1998), which provides top-down attentional modulation and the intermediate/dOT, which provides a visually-based instructive signal to guide calibration of the auditory map (Hyde and Knudsen, 2000; Luksch et al., 2000). Both are anatomically sparse and unlikely to be dominant contributors. space-specific neurons do receive lateral connections from GABAergic neurons within ICX. The inputs to toric spines we observed, however, more closely resemble asymmetric glutamatergic synapses.

To our knowledge, this is the first report of a toric postsynaptic structure. Presynaptic perforations described extensively in the literature are cytoplasmically contiguous (Sorra et al., 1998). In contrast, at least some tori in our SBEM volume were interrupted by plasma membranes that appeared as sites of contact between two “arms” of the same spine (Fig. 9*A*,*B*). The simplest interpretation is these represent enduring adhesions between filopodial extensions emanating from the same base structure. These could be maintained or later converted to cytoplasmic continuities via anastomosis. Regardless of the developmental mechanics, the prevalence and functional properties of contact sites versus cytoplasmic continuities is expected to significantly impact current flow during synaptic stimulation.

The morphologies of ICX neurons from 35- to 40-d old juveniles labeled *ex vivo* and adult space-specific neurons labeled *in vivo* were qualitatively similar with a few clear differences: (1) juvenile cells often expressed a mix of atypical (branched and toric-like) and typical spines whereas adult cells were dominated by either toric (Type I) or typical (Type II) spines, but never both; (2) juvenile cells appeared smaller; and (3) juvenile cells sometimes appeared ciliated. We consider three interpretations: *ex vivo* artifact, unrelated cell type or developmental age.

Preparation of acute brain slices can induce changes in dendritic morphology in comparison to the *in vivo* condition as reflected in perfusion-fixed tissue (Fiala et al., 2003). In this scenario, toric-like spines would represent the slice-induced disassembly of fully-formed toric spines. We think this is highly unlikely due to the number of neuropil elements involved in such a disassembly, specifically the high density of intertwining axons. Whole cell recordings in juvenile slices were targeted under visual guidance to the largest somatic profiles in ICX, the exact location of retrogradely labeled toric space-specific neurons in adults. That the measured size of juvenile cells was smaller is expected from the differential impact of immersion versus perfusion fixation. Specifically, measurements of three neuron types in primate hippocampus indicate that apparent soma volume is 1.36–2.7 times larger in perfusion-fixed than immersion-fixed tissue (Lavenex et al., 2009). On average, our adult cells (perfused brain) were 2.42 (volume) and 1.81 (surface area) times as large as our juvenile cells (immersed slices). Further, the one morphologic attribute not subject to differential shrinkage, number of primary dendrites, was the same in juveniles (4.5) and adults (4.7). In summary, the most straightforward interpretation is that the observed juvenile cell types represent developmental precursors of adult cell types.

Lesion reconstruction (Brainard and Knudsen, 1993), anatomic (Feldman and Knudsen, 1997; DeBello et al., 2001), and pharmacological (Feldman et al., 1996; Zheng and Knudsen, 1999) experiments pinpoint space-specific neurons as the cellular locus of learning (Knudsen, 2002). This raises the question of whether toric spines have a role in experience-dependent synaptic plasticity (DeBello et al., 2014). We found that toric spines are conspicuously well structured to be hubs for microstructural change: of all unmyelinated axons passing within 2 μm of TS1 and TS7, only ∼16% were synaptically connected. Consequently, the number of different wiring diagrams that could be achieved by biologically plausible, small-scale dendritic extensions is very large (Stepanyants et al., 2002; Stepanyants and Chklovskii, 2005). We propose a model in which toric spines actively search for axonal inputs in their local environment, make “tester” synapses, then stabilize or eliminate these on the basis of efficacy in firing the postsynaptic cell (DeBello, 2008). This would provide a mechanism for pattern detection within a complex array of input sources, and the means to adapt connectivity to changing experiential needs. The high degree of cytoskeletal dynamics implicit in this model could also explain the broad spectrum of observed morphologies, with each spine stabilized not by genetic programs but by chance encounters of its extensions within a local, yet information diverse, axonal milieu.

## Synthesis

Reviewing Editor: Darcy Kelley, Columbia University Decisions are customarily a result of the Reviewing Editor and the peer reviewers coming together and discussing their recommendations until a consensus is reached. When revisions are invited, a fact-based synthesis statement explaining their decision and outlining what is needed to prepare a revision will be listed below. The following reviewer(s) agreed to reveal their identity: Catherine Carr, Kristen Harris.

This manuscript constitutes a beautiful new description of “toric” dendritic spines in the barn owl found on a subtype of neurons in the inferior colliculus. The neurons are in a region characterized by experience-dependent plasticity, and the authors hypothesize that these spines, which have numerous active sites, are a locus of sensory integration. The strengths of the work are that is very elegant, combining serial block face EM reconstructions of several spines, and super-resolution optical microscopy. Ex vivo slice recordings were used in owlets to begin to analyze properties of synapses on the space-specific neurons that host these toric spines. The images and reconstructions are excellent, and the descriptions and findings are solid. A significant weakness is that the authors have not yet shown what features distinguish these from other neurons in the external nucleus. One assumes this is the next step. A Pena and Konishi Science paper where Fig. 1B shows a labeled space-specific neuron, characterized with whole cell recording and it might be relevant to mention whether or not this example had especially large and complex spines. There are also several issues that should be addressed as outlined below. Some comments are also offered that would make the paper more readable for the uninitiated.

Major issues:

1) One of the most interesting outcomes is the high number of independent inputs occurring along the finger-like projections from these dendritic protrusions. However, they are certainly not unique in the central nervous system where in many brain regions (even cerebellum, not cited) branched dendritic spines have been noted to have as many as 5 unique axonal inputs in serial EM reconstructions. In hippocampus, and thalamus, the number of branches might be a bit lower than reported for these 'toric' spines, but nevertheless, branched spines have been repeatedly found to have multiple axonal inputs. Yet the authors set us up to believe these may be unique and wait until the discussion to reveal that in fact there are other places where similar branching with multiple inputs occur. Rather than setting it up in the opening paragraph as seemingly unique by stating they have discovered an alternative processing motif, it would be a better setup to start with wanting to understand the space specific network and then to use these multiple approaches to understand the outcomes. In fact, it is more interesting to show this system as not unique, but representative of the branched motif, except in instances like the 'thorny' excrescence where the multiple active zones are with just a few inputs and constitute the detonator synapse motif.

2) Anther concern centers on the issue of 'holes' in the toric spines. Indeed, when viewed in STED some of the spines appear to have holes, rather a spy-glass appearance. However, for all the data that is shown in the paper, and the 3D reconstructions presented, the electron microscopy suggests that these so-called holes are instead fingers emerging from the spines that come into close apposition with many axons passing between them. The authors indicate that continuities occurred between the cytoplasm at the tips of those 'fingers' in about 30% of the spines but everywhere that they refer to 'holes' they indicate as “data not shown”. If indeed holes penetrate the toric endings to these highly branched protrusions, the serial EMs must be shown. Also needed are pictures that would convince us that gap junctions (mentioned also as “data not shown”) could be distinguished in the serial block face images. The name “toric” implies a donut shape with an actual hole in the middle. If that name derives only from the STED images and not the higher resolution 3D reconstructions from serial block face EM, it is a misnomer. Such a misnomer does not detract from the interesting findings regarding the local integration motif. What is needed are several pictures, movies through the serial EMs, and annotated images showing exactly how those fingers come into apposition and that they have cytoplasmic continuity and/or form a gap junction as mentioned.

3) The integrative capacity of these local protrusions will be of interest to a broader audience that those who are familiar with the anatomical abbreviations. It would be easier to read if abbreviations were substantially reduced. SSN - would read much better as 'Space-specific neurons' every where they are mentioned. ITD, ILD, - would read better if spelled out everywhere (except in the figures perhaps) throughout the text. TE for thorny excrescences is a non-standard abbreviation that occurs just a few times and is not easy to remember. Eliminating every abbreviation except those that are truly standard in the broad literature, would improve readability.

4) Page 17: “a situation of functional balance”. Balanced integration needs a more complete definition since the authors do not know which of the synapses are functionally active versus silent (e.g. lacking AMPA receptors, or presynaptic vesicles).

5) Figure 15 is not described in the results section, yet it is perhaps the most critical figure of the paper.

6) Paragraph beginning line 505 (page 21): this paragraph is purely speculative. Models exist where the effective length constant could be tested, and probably should be tested if the authors want to emphasis these toric spines to be independent computational units.

7) The following paragraph beginning line 512 is a bit disjointed. It opens with a review of other simulations is the literature and ends with a statement about their providing the ability for the first time to make high quality patch clamp recordings. The importance for modeling and for basic understanding of the neurons that contain toric spines that deserves a separate paragraph.

8) Paragraph beginning line 574 is difficult to follow: Suggest that the last sentence be moved to the middle, that the parenthetical comment be made into a sentence, and that the conclusion be clarified.

9) Paragraph beginning 602, and especially Lines 617-619: This description belongs in parallel with the various tables. In addition, the last sentence is unclear. Do the authors have some perfusion-fixed owlets where they could ascertain whether the effects were indeed due to slices or developmental differences from adults? This issue deserves attention in the results and methods. Furthermore, the conclusion of the paragraph does not match the argument - namely that the effects are developmental, NOT, ex vivo. Images from a couple of the owlet dendrites from perfusion fixed brain could help make the distinction.

10) Missing from Table 3: Typical single-headed large mushroom spines that contain spine apparatus and might serve to integrate with neighboring small spines (e.g. Chirillo et al., 2019, Sci. Reports).

Minor comments:

1) Page 5, line 63 - word inversion - should read “up to 11 different inputs” not “up to different 11 inputs”.

2) Page 6, line 80: University 1's Institutional... (what does 1's mean here?).

3) Page 8, line 424-425 - Should cite: Mishchenko et al., 2010, Neuron

4) Paragraph beginning on line 441 (page 19) through 463: Figures should be described in order of appearance (e.g. 13 A-F, then 14A-G.

5) Line 494: Check grammar - should probably read: “While morphologically dissimilar from toric spines...

6) Spell out ICCI... Line 533 and elsewhere.

## Author Response

Note: All line numbers refer to the markup version of the revised manuscript, not the clean copy.

Synthesis Statement for Author (Required):

This manuscript constitutes a beautiful new description of “toric” dendritic spines in the barn owl found on a subtype of neurons in the inferior colliculus. The neurons are in a region characterized by experience-dependent plasticity, and the authors hypothesize that these spines, which have numerous active sites, are a locus of sensory integration. The strengths of the work are that is very elegant, combining serial block face EM reconstructions of several spines, and super-resolution optical microscopy. Ex vivo slice recordings were used in owlets to begin to analyze properties of synapses on the space-specific neurons that host these toric spines. The images and reconstructions are excellent, and the descriptions and findings are solid.

We thank the reviewers for thoughtful comments and suggestions, which we believe have substantially improved the manuscript.

A significant weakness is that the authors have not yet shown what features distinguish these from other neurons in the external nucleus. One assumes this is the next step.

A full census of cell types in ICX was beyond the scope of this manuscript; in progress as the reviewers suggest.

A Pena and Konishi Science paper where Fig. 1B shows a labeled space-specific neuron, characterized with whole cell recording and it might be relevant to mention whether or not this example had especially large and complex spines.

This cell appears to host typical spines as well as a few structures that could be large, complex spines. The resolution of that transilluminated micrograph is lower than the images provided in our study, therefore we did not characterize this cell. Our best guess is Type II SSN, consistent with STED results. See lines 326 - 331 in revised manuscript.

There are also several issues that should be addressed as outlined below. Some comments are also offered that would make the paper more readable for the uninitiated.

Major issues:

1) One of the most interesting outcomes is the high number of independent inputs occurring along the finger-like projections from these dendritic protrusions. However, they are certainly not unique in the central nervous system where in many brain regions (even cerebellum, not cited) branched dendritic spines have been noted to have as many as 5 unique axonal inputs in serial EM reconstructions. In hippocampus, and thalamus, the number of branches might be a bit lower than reported for these 'toric' spines, but nevertheless, branched spines have been repeatedly found to have multiple axonal inputs. Yet the authors set us up to believe these may be unique and wait until the discussion to reveal that in fact there are other places where similar branching with multiple inputs occur. Rather than setting it up in the opening paragraph as seemingly unique by stating they have discovered an alternative processing motif, it would be a better setup to start with wanting to understand the space specific network and then to use these multiple approaches to understand the outcomes. In fact, it is more interesting to show this system as not unique, but representative of the branched motif, except in instances like the 'thorny' excrescence where the multiple active zones are with just a few inputs and constitute the detonator synapse motif.

These insights are very helpful. The manuscript has been revised to clarify what is and is not new about toric spines: (a) their morphology appears unprecedented (b) the innervation of complex spine types by more than one axon (now termed 'integrative capacity' adopted from reviewer language below) is not. We now include references to integrative capacity in cerebellum, hippocampus and thalamus. In these studies, we found statements and some data consistent with multiple axon convergence (lines 565-586). That said, quantitative demonstration was, in our view, not as extensive as what is shown in our manuscript. If the reviewers are aware of other prior publications that convincingly demonstrate multiple axon convergence and that they believe we should reference, we would be grateful if they brought them to our attention.

Nonetheless, we agree with the main thrust of this concern: while the integrative capacity of toric spines is firmly established and the highest (up to 11 axons per spine) thus far demonstrated, the more important implication is that this motif is likely not unique - indeed it may be more common than currently appreciated. This theme is reflected throughout the revised manuscript and highlighted in the Discussion (lines 488-506; lines 565-586). The introduction was also modified to remove mention of 'discovery' of convergence.

2) Anther concern centers on the issue of 'holes' in the toric spines. Indeed, when viewed in STED some of the spines appear to have holes, rather a spy-glass appearance. However, for all the data that is shown in the paper, and the 3D reconstructions presented, the electron microscopy suggests that these so-called holes are instead fingers emerging from the spines that come into close apposition with many axons passing between them. The authors indicate that continuities occurred between the cytoplasm at the tips of those 'fingers' in about 30% of the spines but everywhere that they refer to 'holes' they indicate as “data not shown”. If indeed holes penetrate the toric endings to these highly branched protrusions, the serial EMs must be shown. Also needed are pictures that would convince us that gap junctions (mentioned also as “data not shown”) could be distinguished in the serial block face images. The name “toric” implies a donut shape with an actual hole in the middle. If that name derives only from the STED images and not the higher resolution 3D reconstructions from serial block face EM, it is a misnomer. Such a misnomer does not detract from the interesting findings regarding the local integration motif. What is needed are several pictures, movies through the serial EMs, and annotated images showing exactly how those fingers come into apposition and that they have cytoplasmic continuity and/or form a gap junction as mentioned.

These are important points we failed to make clear. Notwithstanding the order of presentation in the manuscript, 'holes' were discovered in the SBEM volume. Based on apparent continuity of DAB label they appear as topological holes in 3D models, and by this definition holes occur in about 30% of spines (the larger, more complex ones). On close inspection some holes were bounded by filopodial arms that touched. The membrane appositions at these touch points resemble synaptic junction / tight junction / gap junction i.e. ~20 nm cleft which appears as a DAB continuity in most image sections. We also found one clear instance of a hole bounded entirely by cytoplasmic continuity - no membrane appositions. Importantly, the dark cytoplasmic label in combination with narrow tubular build and twisted morphology made tracking cytoplasmic continuity throughout entire spines fraught with ambiguity. Thus we did not quantify the prevalence of touches (DAB continuity with apparent tight junction) vs true cytoplasmic continuities.

To address this concern we added a new figure, Fig. 9, showing one example of each.

3) The integrative capacity of these local protrusions will be of interest to a broader audience that those who are familiar with the anatomical abbreviations. It would be easier to read if abbreviations were substantially reduced. SSN - would read much better as 'Space-specific neurons' everywhere they are mentioned. ITD, ILD, - would read better if spelled out everywhere (except in the figures perhaps) throughout the text. TE for thorny excrescences is a non-standard abbreviation that occurs just a few times and is not easy to remember. Eliminating every abbreviation except those that are truly standard in the broad literature, would improve readability.

We eliminated from the text all SSN and TE abbreviations and most ITD and ILD. Kept 'ITD-ILD' at one spot in discussion after defining earlier in paragraph. Kept brain regions acronyms e.g. 'ICCls' for lateral shell of the central nucleus of the inferior colliculus. Kept most acronyms in figure legends.

4) Page 17: “a situation of functional balance”. Balanced integration needs a more complete definition since the authors do not know which of the synapses are functionally active versus silent (e.g. lacking AMPA receptors, or presynaptic vesicles).

Revised this paragraph and figure legend to refer solely to 'anatomical balance.'

5) Figure 15 is not described in the results section, yet it is perhaps the most critical figure of the paper.

Moved to Figure 5 to better place in context of Results.

6) Paragraph beginning line 505 (page 21): this paragraph is purely speculative. Models exist where the effective length constant could be tested, and probably should be tested if the authors want to emphasis these toric spines to be independent computational units.

NEURON simulation has proven more difficult than anticipated. In the third paragraph of Discussion we state this and also removed the phrase 'independent computational subunit' while preserving the idea that toric spine are the first stage of integration. As the reviewers indicate, that could or could not be independent of processing at later stages.

7) The following paragraph beginning line 512 is a bit disjointed. It opens with a review of other simulations is the literature and ends with a statement about their providing the ability for the first time to make high quality patch clamp recordings. The importance for modeling and for basic understanding of the neurons that contain toric spines that deserves a separate paragraph.

Revised and moved to separate paragraph (page 4 of Discussion).

8) Paragraph beginning line 574 is difficult to follow: Suggest that the last sentence be moved to the middle, that the parenthetical comment be made into a sentence, and that the conclusion be clarified.

Revised as suggested.

9) Paragraph beginning 602, and especially Lines 617-619: This description belongs in parallel with the various tables. In addition, the last sentence is unclear. Do the authors have some perfusion-fixed owlets where they could ascertain whether the effects were indeed due to slices or developmental differences from adults? This issue deserves attention in the results and methods. Furthermore, the conclusion of the paragraph does not match the argument - namely that the effects are developmental, NOT, ex vivo. Images from a couple of the owlet dendrites from perfusion fixed brain could help make the distinction.

The reviewers are correct that perfusion-fixed tissue from owlets would resolve this issue. Unfortunately, we do not have birds of the right age at this time. The final sentence referred solely to ciliation, not toric vs toric-like appearance. As ciliation is immaterial to the conclusions of the manuscript, that confusing sentence was deleted.

10) Missing from Table 3: Typical single-headed large mushroom spines that contain spine apparatus and might serve to integrate with neighboring small spines (e.g. Chirillo et al., 2019, Sci. Reports).

There are several worthy candidates for inclusion in Table 3. We included only typical spines and TEs (closest morphology to toric spines) as the bare minimum context. A full compare-and-contrast across known and emerging spines types could merit a review article.

Minor comments:

1) Page 5, line 63 - word inversion - should read “up to 11 different inputs” not “up to different 11 inputs”.

Done.

2) Page 6, line 80: University 1's Institutional... (what does 1's mean here?).

The journal requires us to remove information that could identify the authors. '1' will be replaced with a name if the manuscript is published.

3) Page 8, line 424-425 - Should cite: Mishchenko et al., 2010, Neuron

Done. This is a key reference.

4) Paragraph beginning on line 441 (page 19) through 463: Figures should be described in order of appearance (e.g. 13 A-F, then 14A-G.

Done.

5) Line 494: Check grammar - should probably read: “While morphologically dissimilar from toric spines...

Done.

6) Spell out ICCI... Line 533 and elsewhere.

The large majority of acronyms are now spelled out. We kept the anatomical acronyms to avoid word clutter, but defer to reviewer expertise and journal standards on this point.
